# Greenland Norse walrus exploitation deep into the Arctic

**DOI:** 10.1126/sciadv.adq4127

**Published:** 2024-09-27

**Authors:** Emily J. Ruiz-Puerta, Greer Jarrett, Morgan L. McCarthy, Shyong En Pan, Xénia Keighley, Magie Aiken, Giulia Zampirolo, Maarten J. J. E. Loonen, Anne Birgitte Gotfredsen, Lesley R. Howse, Paul Szpak, Snæbjörn Pálsson, Scott Rufolo, Hilmar J. Malmquist, Sean P. A. Desjardins, Morten Tange Olsen, Peter D. Jordan

**Affiliations:** ^1^ Section for Molecular Ecology and Evolution, Globe Institute, Faculty of Health and Medical Sciences, University of Copenhagen, Øster Farimagsgade 5-7, DK-1353 Copenhagen K, Denmark.; ^2^ Arctic Centre and Groningen Institute of Archaeology, Faculty of Arts, University of Groningen, PO Box 716, NL-9700 AS Groningen, Netherlands.; ^3^ Department of Archaeology and Ancient History, Lund University, Helgonavägen 3, 223 62 Lund, Sweden.; ^4^ Palaeobiology Section, Canadian Museum of Nature, P.O. Box 3443, Station D, Ottawa, Ontario K1P 6P4, Canada.; ^5^ The Bureau of Meteorology, The Treasury Building, Parkes Place West, Parkes, ACT 2600, Australia.; ^6^ Section for GeoGenetics, Globe Institute, University of Copenhagen, Øster Voldgade 5-7, 1350 Copenhagen K, Denmark.; ^7^ Inuit Heritage Trust Inc., 2425 Abe Okpik, Iqaluit, Nunavut X0A 2H0, Canada.; ^8^ Department of Anthropology, Trent University, 1600 West Bank Drive, Peterborough, Ontario K9L 0G2, Canada.; ^9^ Faculty of Life and Environmental Sciences, University of Iceland, Askja, Sturlugata 7, 101 Reykjavik, Iceland.; ^10^ Icelandic Museum of Natural History, Suðurlandsbraut 24, 108 Reykjavík, Iceland.; ^11^ Global Station for Indigenous Studies and Cultural Diversity (GSI), GI-CoRE, Hokkaido University, Sapporo, Japan.

## Abstract

Walrus ivory was a prized commodity in medieval Europe and was supplied by Norse intermediaries who expanded across the North Atlantic, establishing settlements in Iceland and Greenland. However, the precise sources of the traded ivory have long remained unclear, raising important questions about the sustainability of commercial walrus harvesting, the extent to which Greenland Norse were able to continue mounting their own long-range hunting expeditions, and the degree to which they relied on trading ivory with the various Arctic Indigenous peoples that they were starting to encounter. We use high-resolution genomic sourcing methods to track walrus artifacts back to specific hunting grounds, demonstrating that Greenland Norse obtained ivory from High Arctic waters, especially the North Water Polynya, and possibly from the interior Canadian Arctic. These results substantially expand the assumed range of Greenland Norse ivory harvesting activities and support intriguing archaeological evidence for substantive interactions with Thule Inuit, plus possible encounters with Tuniit (Late Dorset Pre-Inuit).

## Teaser

European demand for walrus ivory drove Greenland Norse interactions with Arctic Indigenous peoples.

## INTRODUCTION

The Arctic experienced the dispersal and contraction of several major cultural groups during the Medieval Warm Period (ca. 950 to 1250 CE). The maritime-adapted Thule Inuit expanded eastward from Alaska across Arctic Canada (Inuit Nunangat) and into Greenland (Kalaallit Nunaat) as early as the 13th century CE, resulting in encounters, displacement, and eventual replacement of the Tuniit (Late Dorset Pre-Inuit) culture (*1*–*5*). Over the same period, groups with primary cultural and genealogical ties to Iceland and Scandinavia (collectively defined here as the Greenland Norse) settled in southwestern Greenland, explored surrounding regions, and established an export-led economy that supplied walrus ivory back to trade centers in Europe (*3*–*6*). Key historical questions about the Greenland Norse (ca. 985 to 1450 CE) revolve around (i) the nature and extent of Norse encounters with the Tuniit and Thule Inuit, (ii) whether organized trade in walrus ivory emerged between groups, and (iii) if so, where, when, and why such interactions occurred. These issues are important to resolve, not least because meetings between the European Norse and Indigenous North Americans represent the first “full circle” reconnection of the two major branches of Pleistocene human dispersals out of Africa (*1*, *7*–*9*). To address these questions, we genetically sourced 31 cultural artifacts made from Atlantic walrus (*Odobenus rosmarus rosmarus*) back to specific Arctic hunting grounds. These objects were central to the Norse ivory trade and were recovered from Greenland Norse settlements and several major European trade hubs (see table S1). The results were contextualized with experimental insights into Greenland Norse seafaring capabilities (*10*–*16*). Our goal was to evaluate the extent to which the Greenland Norse obtained ivory via direct hunting versus exchange with Tuniit or Thule Inuit groups and the likely locations and timings of the walrus hunts and possible intercultural encounters.

From the late 9th to the mid-14th century CE, walrus ivory was exchanged into European trade and production centers via Norse intermediaries who operated across the North Atlantic. The opening phases of commercial Norse walrus hunting were probably unsustainable, starting in Fennoscandia, then spreading to Iceland in the early ninth century, where the local walrus population was eventually extirpated; the Norse then expanded into Greenland and established permanent settlements (*17*–*19*). Here, the Greenland Norse communities (ca. 985 to 1450 CE) gained a virtual monopoly on ivory supplies into Europe from the early 12th to the mid-14th century, with exports into Europe peaking around 1250 CE (*17*, *20*). However, it is unclear whether all the ivory passing through the Greenland Norse settlements was directly hunted by Norse, or partly, or even entirely, exchanged with Arctic Indigenous groups, as both Tuniit (*21*) and the expanding Thule Inuit (*22*) were also present in adjacent areas of Arctic Canada and northwest Greenland over the same broad historical interval. The small Greenland Norse communities may have struggled to mount long-range hunting expeditions, making trade with other Arctic hunting groups an attractive alternative. Conversely, the high commercial value of ivory potentially encouraged the Greenland Norse to prioritize walrus hunting over other branches of their economy, including farming (*23*). The Greenland Norse were certainly aware of Thule Inuit and Tuniit groups and may have used initial encounters to explore opportunities for more formalized ivory exchange, though what the Norse could offer in return remains unclear (*24*, *25*). Some Greenland Norse contact with the Tuniit does seem likely despite the scarcity and ambiguity of archaeological evidence, especially considering the 300 years of temporal overlap in the Baffin Bay and Davis Strait area (see Supplementary Text). Indications of possible Norse-Tuniit encounters were also discovered in the Smith Sound region, located between Ellesmere Island and Northwest Greenland, including a fragment of a brass pot recovered from a reliably dated Tuniit context (*26*). There is more substantive archaeological evidence for considerable spatiotemporal overlap between Thule Inuit and Greenland Norse, including indications that the expanding Thule Inuit may eventually have hunted marine mammals in Disko Bay (*26*) and occupied seasonal sites as far south as Sandhavn, located quite close to the Eastern Settlement of the Greenland Norse (*27*).

To better understand the Arctic dimensions of the Greenland Norse ivory harvesting and trade networks, including the location and timing of intercultural encounters, we defined three contrasting Norse exploitation scenarios. These were evaluated empirically with high-resolution genomic sourcing methods to understand changing patterns of Norse walrus exploitation: scenario 1: Direct Norse Exploitation—written sources mention annual summer walrus hunting expeditions to the *Norðrsetur*, an ill-defined coastal area located north of the Western Settlement (*5*, *28*–*31*). While there is no direct archaeological evidence that the Greenland Norse possessed specialized walrus hunting equipment, they certainly had directly relevant hunting experience from Iceland and Fennoscandia, and probably used lances to target walrus at historically documented haul-out sites (*25*, *32*–*34*); scenario 2: Norse-Indigenous Trade—historical records confirm that the Greenland Norse swiftly acquired knowledge of the wider regional geography, including the presence of other cultural groups. While initial encounters with Tuniit or Thule Inuit may have involved avoidance and occasional skirmishes, formalized ivory trading could have emerged thereafter (*11*, *13*); scenario 3: Evolving Strategies—the Norse may have hunted local walrus upon arriving in Greenland, but were then forced to visit ever more distant hunting grounds as local stocks were depleted. Such voyages would have increased the likelihood of encounters, especially if other Arctic Indigenous groups were hunting similar resources in the same areas, perhaps encouraging a shift from direct Norse acquisition to some form of exchange relations. If more formalized trading relations did somehow emerge, they would represent some of the earliest steps toward circumpolar “globalization,” a process that would eventually define later historical periods, including expansive culture contacts, intensive trade networks, and the market-driven exploitation of the Arctic’s natural resources by distant polities and urban consumption centers.

## RESULTS

### Ancient DNA analyses support sourcing of walrus artifacts back to specific hunting grounds

**Fig. 1.**
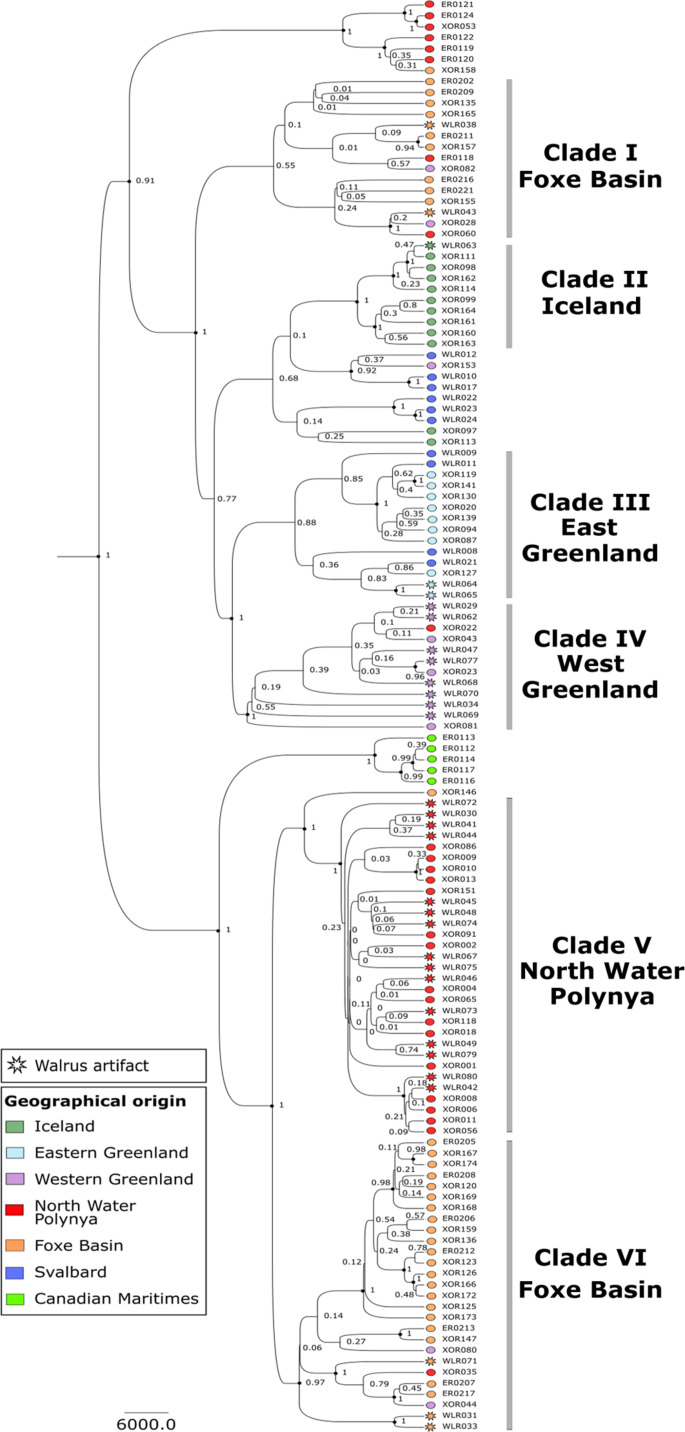
Genetic sourcing of traded artifacts back to specific walrus stocks. The Bayesian phylogeny includes walrus mitogenomes from 100 biological samples and 31 cultural artifacts. These biological samples were obtained from a wide range of geographic locations and chronological periods to reconstruct genetic diversity and stock locations at the time of the Norse Greenland settlements. Our results confirm that distinct walrus stocks were located in specific locations (Fig. 2 and table S1). This combined approach enabled the walrus artifacts recovered from trade and production centers in Europe and the main Greenland Norse settlements to be genetically sourced back to specific walrus stocks and particular Arctic hunting grounds (Fig. 2 and table S2). The phylogeny is rooted against the Pacific walrus (not shown). Black circles denote nodes with >90% posterior support. Figure: E.J.R.-P. and coauthors.

**Fig. 2.**
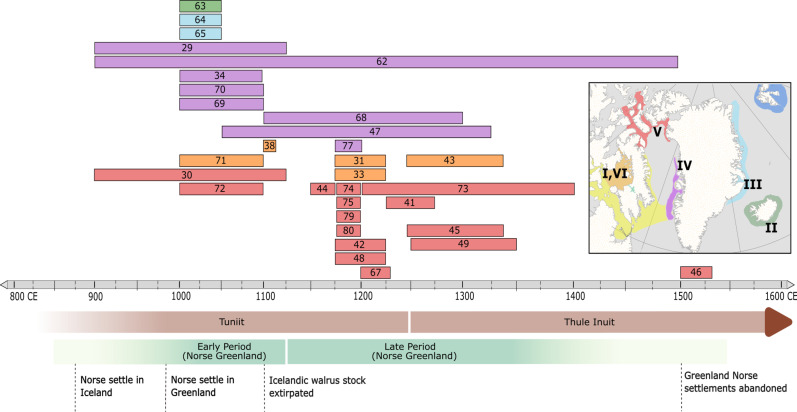
Patterns of Greenland Norse walrus exploitation shifted over time. Dated walrus artifacts sourced to different Arctic hunting grounds (*n* = 31). Artifacts were allocated chronologically to either the Early Period or Late Period of Norse Greenland (before/after 1120 CE), plus allocated more specific date ranges if available (see table S2). Numbered bands are individual artifacts (for full provenance information, see table S2, using WLR0 + sample number); the colors match specific walrus stocks in the inset map (right). The main trends in exploitation indicate the following: (i) initial Norse harvesting focused on stocks near Iceland (II and III); (ii) Early Period Greenland Norse mainly harvested the local stock (IV) located near to their main settlements; and (iii) the increasing importance of High Arctic walrus stocks in the Late Period, especially the North Water Polynya (V), and also Foxe Basin (I and VI). Last, the following should also be noted (iv) even in the Early Period, Greenland Norse were also acquiring some ivory from distant walrus stocks (I, VI, and V); and (v) Late Period harvesting continued at the local stock (IV). The expanding geographic range of Greenland Norse walrus harvesting likely led to initial Tuniit encounters in several different areas; more definitive interactions with expanding Thule Inuit populations probably focused on the North Water Polynya (V). No artifacts were sourced to the Canadian Maritimes or to Svalbard (see table S2 and the main text). Figure: E.J.R.-P. and coauthors.

We used ancient DNA analyses to reconstruct how the Greenland Norse harvested walrus ivory from different Arctic hunting grounds. Previous isotopic and mitogenomic sourcing efforts have identified a chronological shift in Norse walrus exploitation across the North Atlantic. The process starts with a focus on eastern stocks located closer to Fennoscandian waters, and then shifts over to western walrus populations, although the role of more specific hunting grounds remains uncertain (*17*–*19*). To resolve this gap in knowledge, we used Bayesian phylogeographic analyses of mitogenomes from 100 biological walrus samples and 31 dated cultural artifacts, allowing us to assign each traded walrus artifact back to a specific walrus stock (Fig. 1; see also Materials and Methods). The biological walrus samples were obtained from different Arctic locations and relevant chronological intervals, representing the genetic diversity and walrus stock locations at the time of the Greenland Norse settlements (ca. 985 to 1450 CE) (see table S1). The targeting of both ancient and historical samples to build the phylogeny significantly improves the resolution of previous studies and also resolves concerns that walrus stocks may have shifted or merged due to later habitat disruptions and industrial-scale harvesting (*17*). The 31 walrus artifacts were all recovered from Norse sites in Europe [see table S2; data published previously, (*17*)]. In general, the Greenland Norse shipped walrus ivory out to European markets in the form of tusks left attached to the front portions of the walrus skull, i.e., the rostrum. We assume that these “packages” were broken open relatively soon after arriving into European workshops to extract the precious ivory and produce the valuable objects required for elite consumption and display. In this way, we assume that the distinctive bone production waste serves as a direct proxy for wider ivory trade networks. This approach enabled us to genetically track the Greenland Norse ivory trade networks from European centers all the way back to specific Arctic hunting grounds and also to examine the extent to which spatial patterns of Norse walrus exploitation had shifted over time (Fig. 2).

### Early Norse ivory exploitation targeted local stocks

To understand the chronology of walrus exploitation, we divide the history of Norse Greenland (ca 985 to 1450 CE) into an “Early Period” before 1120 CE and a “Late Period” after this date, following Star *et al.* (*17*) (Fig. 2; see Materials and Methods). We sourced 11 artifacts assigned chronologically to the Early Period. Our results indicate that the Norse initially exploited stocks closest to their settlement areas: first in Iceland and East Greenland (or Greenland Strait, north of Iceland), and then in the Disko Bay region after the Norse settlements in Greenland had been established (Fig. 2). Three objects recovered from Sigtuna, Sweden, originated in the now extinct Icelandic stock (clade II, WLR063) and the East Greenland stock (clade III, WLR064 and WLR065), while one walrus artifact from Dublin, Ireland, can be traced to the West Greenland stock (clade IV, WLR029). Similarly, two artifacts from Garðar (Igaliku) in the Eastern Settlement of Norse Greenland also appear to originate from the local West Greenland walrus stock (clade IV, WLR69 and WLR70). Two artifacts (from Dublin, Ireland, and Garðar, Greenland) are both assigned to the Early Period but appear to be made from ivory originating in the distant North Water Polynya, which is located between Northwest Greenland and Northeast Canada (clade V, WLR030 and WLR072). Last, and with slightly lower phylogenetic support, two further Early Period objects recovered from Trondheim, Norway, and Garðar, Greenland, can be sourced to either the Foxe Basin or to the West Greenland stock (clade I, WLR038 and clade VI, WLR071). Overall, our results from the Early Period confirm that walrus exploitation, including the export of ivory back to distant European consumers, supported the economy of the Greenland Norse communities from their establishment. Last, these initial Greenland Norse harvesting patterns appear to have formed a logical stepwise geographic expansion of walrus exploitation into new areas, probably using similar hunting strategies. Before this, Norse harvesting efforts had focused on Fennoscandia, and then shifted out to Icelandic waters until local stocks were overexploited (*11*, *19*, *23*, *25*, *35*, *36*).

### Greenland Norse obtained walrus ivory from High Arctic hunting grounds

We sourced 20 walrus artifacts assigned chronologically to the Late Period (Fig. 2). Most of these date to the mid-12th to late 13th century, an interval that corresponds to both major socio-political transformations within Scandinavia plus the peaking of demand for walrus ivory across European trade networks (*20*, *36*, *37*). Our results indicate a major geographic shift in walrus exploitation patterns: As the Greenland Norse sought to maintain their supply of ivory to European markets, they appear to have relied increasingly on harvesting ivory from more distant hunting grounds located much deeper into the High Arctic. We sourced 14 artifacts—close to half of those in our study—back to the North Water Polynya walrus stock (clade V), which centers around the marine-ecological “hot spot” of the Pikialasorsuaq (*38*). In addition, we more tentatively sourced three further artifacts back to the Foxe Basin stock (allocated to clades I and VI: WLR031 and WLR033; London, WLR043; Bergen). To exploit these much more distant stocks, the Greenland Norse must either have been mounting their own long-range hunting expeditions from their main base settlements, voyaging deep into High Arctic waters, or were meeting and trading with Arctic Indigenous groups who did the primary hunting of these more distant walrus stocks. However, it also appears that even in the Late Period, the Greenland Norse were still able to harvest at least some ivory quite close to their main settlements, with two artifacts from Schleswig (WLR068) and Kyiv (WLR077) originating in the West Greenland walrus stock (clade IV). Last, four further artifacts assumed to date to the general interval of the Greenland Norse settlements (ca. 985 to 1450 CE), albeit with some chronological uncertainties (see table S2), were also sourced: Two originated in the West Greenland stock (clade IV, WLR047 and WLR068), and two originated in the North Water Polynya stock (clade V, WLR046 and WLR048).

### Greenland Norse seafaring capacities potentially supported High Arctic expeditions

**Fig. 3.**
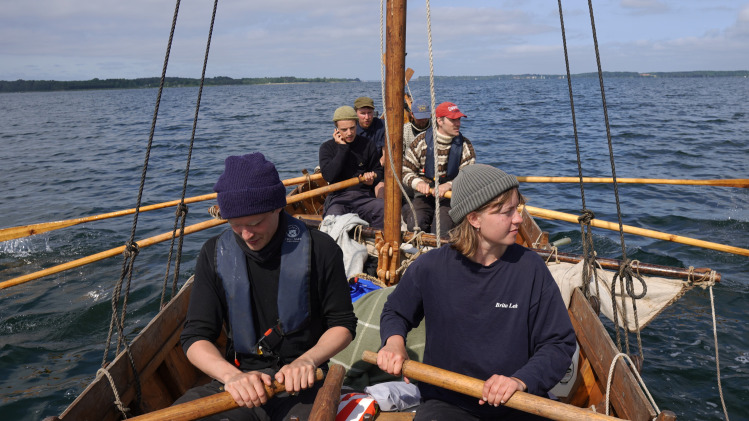
Experimental insights into Greenland Norse seafaring capabilities: example of a “smaller” vessel (with oars and sail). This is a Norwegian fyring during sea trials. Note the very limited space for cargo (Roskilde Fjord, Denmark, June 2023). Photo: G.J.

**Fig. 4.**
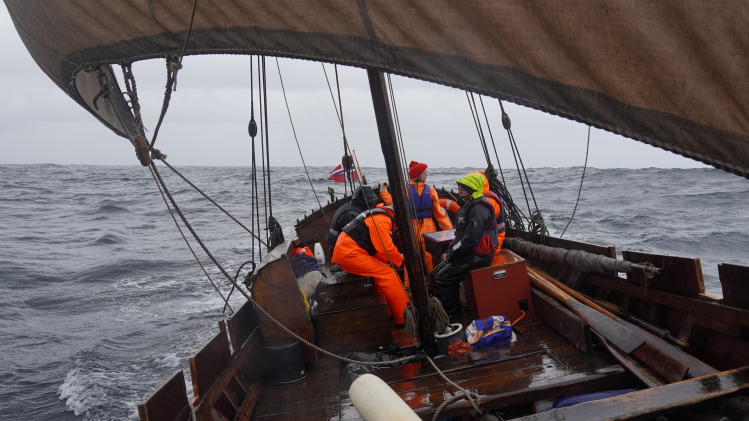
Experimental insights into Greenland Norse seafaring capabilities: example of a larger expeditionary sailing vessel. This is a Norwegian fembøring, a direct descendant of the Norse clinker tradition used in Greenland (Vestfjord, northern Norway, May 2022). Only these larger sailing ships, owned and sponsored by richer farmers and elites, would have been capable of reaching the North Water Polynya during single-summer expeditions. One major risk was becoming trapped in the expanding late-summer pack ice, forcing the crew to overwinter en route, as evidenced by the Kingittorsuaq runestone (Fig. 5) carved during the Spring, and dating to ca. 1250 to 1300 CE (see Supplementary Text). Photo: G.J.

**Fig. 5.**
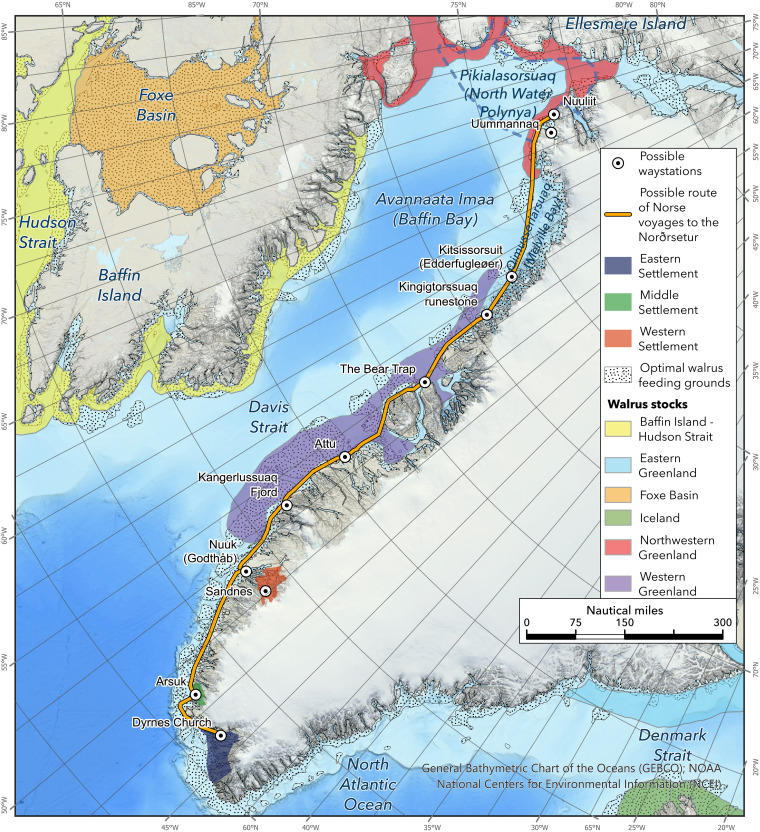
Postulated south-north maritime corridor linking the permanent Greenland Norse settlements into Northwest Greenland and High Arctic Canada. This schematic map depicts the location of the main Norse settlements, primary navigation routes, and likely stopping points in relation to major walrus hunting grounds (for further details, see Supplementary Text). Map: G.J.

The substantial geographic expansion of walrus ivory harvesting efforts in the Late Period raises a central question: Did the Greenland Norse communities have the seafaring capabilities and motivations required to access the more distant High Arctic walrus stocks located at the North Water Polynya (clade V) and Foxe Basin (clades I and VI)? Greenland Norse had limited seasonal windows available for summer hunting expeditions, probably no more than 10 weeks (see Supplementary Text). Our research suggests that two distinct vessel types were available at the main Norse settlements in southwest Greenland: (i) smaller six-oared boats with a crew of 6 or 7 (Fig. 3 and fig. S1) and (ii) larger “expeditionary” ships carrying crews of 15 to 40 (Fig. 4 and fig. S2). The latter vessels had been used on exploration voyages to Greenland and North America and were owned by wealthier farmers or sponsored by social elites (*25*, *29*, *31*). We estimated sailing times and handling capabilities of these two different classes of vessel using documentary sources and experimental sea trials (see Supplementary Text). We also reconstructed likely sailing routes to different walrus stocks and identified possible stopping points and overwintering stations (Fig. 5 and table S3). The combined results indicate that the smaller six-oared boats could have been rowed from the Western Settlement as far as the Qeqertarsuup Tunua (Disko Bay). However, it was also clear that longer-range expeditions to the Pikialasorsuaq (North Water Polynya) could only have been possible with the larger expeditionary sailing ships capable of making the 2- to 3-day crossing from Kitsissorsuit (Edderfugleøer) to Innaanganeq (Cape York). Deploying the larger ships, the Qeqertarsuup Tunua (Disko Bay) region could probably have been reached within 6 to 10 days. However, sailing on as far as the Pikialasorsuaq (North Water Polynya) hunting grounds (clade V) would have taken approximately 30 days in total. We estimate that the return journey would have been shorter due to more favorable weather conditions later in the summer, taking approximately 15 days (table S3). Assuming Norse expeditions departed the Western Settlement in early to mid-June, they would have reached the Pikialasorsuaq (North Water Polynya) in mid-July, giving the crews 2 to 4 weeks to acquire ivory, before departing back to the Norse settlements, and arriving home in late August as the autumn storms closed in. As the Norse lacked Thule Inuit toggling-harpoon technology to hunt walrus in the open sea, it is likely that the animals were targeted at haul-out sites and then killed with lances, with several hundred animals possibly harvested and processed during each expedition (*6*, *25*, *39*–*41*) (see Supplementary Text). Depending on the precise size of Norse crews and their vessels, the harvesting process might have been completed within one sustained session at a single haul-out site. More likely, the crews undertook multiple short-range harvesting trips from a more central base camp out to surrounding walrus haul-out sites. Some archaeological features, including the “Bear Trap” (fig. S3), hint at complex mobility strategies involving the construction of central storage facilities (*25*, *32*). The hide and tusks of a large adult walrus weigh approximately 50 kg (*25*). Depending on whether crews prioritized ivory, or a combination of tusks and hides, a six-oared boat with a cargo capacity of 1 ton could only transport approximately 20 sets of hides and tusks, while one of the larger vessels could transport between 85 and 400 sets, assuming a cargo capacity range of 4.5 to 21 tons (*29*, *42*, *43*) (see Supplementary Text).

## DISCUSSION

**Fig. 6.**
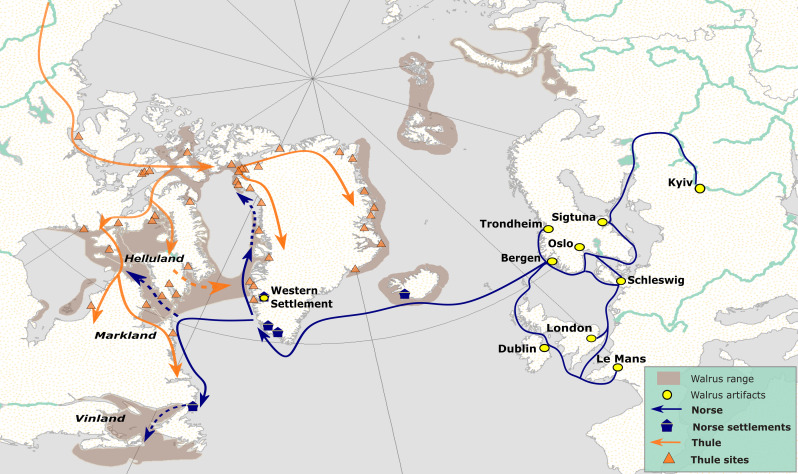
Early circumpolar globalization: schematic reconstruction of the Arctic Ivory Road. Shifting walrus exploitation patterns suggest a “domino” model: the Norse systematically depleted more accessible walrus stocks to supply the booming European ivory trade; the search for fresh sources of ivory was one factor driving Norse expansion into the Northwest Atlantic, including initial colonization of Iceland, and the establishment of Norse settlements in Southwest Greenland. Exploration of coastal North America (Helluland, Markland, and Vinland) by the Norse likely resulted in initial full-circle encounters with various Indigenous North American groups across a broad “contact” frontier running from the Canadian Maritimes up to the High Arctic. However, most ivory in the Early Period (pre 1120 CE) was coming from the local stock in West Greenland (IV). By the Late Period (after 1120 CE), Greenland Norse communities were mounting regular long-range expeditions to the High Arctic to harvest ivory from the North Water Polynya (Stock V), either via direct hunting, or intercultural trade and exchange, possibly with Tuniit groups, and more probably with the Thule Inuit who were expanding across the Canadian Arctic and into this area. These routine intercultural interactions at the North Water Polynya peoples signal the onset of early circumpolar globalization, with numerous Norse artifacts recovered from Thule Inuit sites dating to this interval. The Greenland Norse may also have ventured deeper into the interior Canadian Arctic waters, or more likely hunted walrus and traded ivory with Arctic Indigenous peoples at intermediate locations (Stocks I,VI). With elite consumption trends in remote European urban centers driving these early full-circle global interactions, our preliminary reconstructions of the emerging Arctic Ivory Road bear interesting parallels with Silk Road that spanned Medieval Eurasia during the same period. Figure: E.J.R.-P. and coauthors.

Application of higher-resolution genetic sourcing methods enabled us to track the Greenland Norse ivory trade back to much more specific Arctic hunting grounds, advancing previous studies (*17*, *19*, *44*). Our results confirm that walrus exploitation was central to the Norse expansion into the Northwest Atlantic, likely encouraging initial exploration and then more permanent settlement of Iceland and Greenland (*20*). Walrus exploitation therefore expanded stepwise into new areas, starting in Fennoscandia, then moving to Iceland, East Greenland, West Greenland, and lastly penetrating the High Arctic. This pattern potentially signals an ecological “domino model” in which the European demand drove relentless overexploitation of more accessible walrus stocks, pushing Norse hunters into ever more remote areas in their search for valuable ivory. While our overall findings confirm this general pattern, we found no evidence of Norse walrus exploitation reaching as far as the waters around Svalbard (Figs. 2 and 6; see Materials and Methods); the primary vector of Norse expansion was into the Northwest Atlantic. In the Early Period, the Greenland Norse mainly targeted local stocks, but by the Late Period, primary harvesting appears to have shifted up to the High Arctic, with efforts focusing on the Pikialasorsuaq (North Water Polynya), and possibly expanding into the waters of the Foxe Basin (Fig. 6).

Returning to our three exploitation scenarios, our combined evidence points to scenario 1 (Direct Norse Harvesting) as the dominant pattern of exploitation in the Early Period. Most artifacts from this period source back to more accessible stocks located within easy reach of the main Icelandic and Greenland Norse settlements. Moreover, both stock locations are far removed from known areas of Tuniit and Thule Inuit settlement, making scenario 2 (Norse-Indigenous Trade) unlikely. However, sporadic encounters and some opportunistic exchange may have occurred during the initial Greenland Norse explorations mentioned above (*45*), possibly involving the Tuniit, whose communities were more widely distributed at this time. These very earliest full-circle encounters between the Norse and Tuniit potentially created an extended “frontier” of initial European-Indigenous encounters, and may predate those associated with the short-lived L’Anse aux Meadows site, which was established by expanding Norse groups in the Canadian Maritimes (Fig. 6, see Supplementary Text). Walrus populations were also located in this area, though none of our artifacts were sourced back to this particular stock (see Fig. 1 and table S2), perhaps suggesting that other factors motivated Norse explorations into this region (see table S2).

In contrast, our results confirm that the Pikialasorsuaq (North Water Polynya) had emerged by the Late Period as the primary location for Greenland Norse ivory harvesting: Tuniit communities operated here until at least 1200 CE, with Thule Inuit groups arriving slightly later. Norse (or Norse-inspired) material culture then appears in Thule Inuit sites dating to the 13th to 14th century CE, with some artifacts recovered from occupations located deep into the Canadian High Arctic (*1*, *12*, *26*, *45*–*47*). One possibility is that Tuniit or Thule Inuit were harvesting ivory at the North Water Polynya and then voyaging south to trade. However, this seems unlikely as Greenland Norse communities were short of metal and other materials that could motivate regular long-distance trading visits by Arctic Indigenous peoples (*25*). In contrast, it was the Greenland Norse who had the greatest incentive to voyage deep into the High Arctic in search of ivory; they also had the seafaring capabilities, and emergent socio-political dynamics may have led elites in Greenland and Norway to sponsor such longer-range harvesting expeditions (see Supplementary Text). Despite these motivations, the Greenland Norse visits to High Arctic hunting grounds were probably occasional rather than annual, especially after the onset of deteriorating weather and sea-ice conditions in the 13th century (*48*). Our research identified narrow seasonal windows, with the longer-range expeditions fraught with risk, generating further task-scheduling conflicts if crews failed to return by the vital hay-making season that provided winter fodder for animals back at the main Greenland Norse settlements (*49*). Despite these challenges, one successful expedition every few years, involving a handful of ships and a few weeks of intense effort, could easily generate the ivory exports of the volumes recorded in historical sources (see Supplementary Text).

We reach the conclusion that scenario 3 (Evolving Strategies) captures the main exploitation patterns in the Late Period, as the combined evidence indicates that Tuniit, Thule Inuit, and Norse groups were all operating around the Pikialasorsuaq (North Water Polynya), targeting the same resources in the same historical period, making routine encounters almost certain and some degree of formalized exchange increasingly likely. Whatever the precise character of these interactions, the Pikialasorsuaq (North Water Polynya) can now be identified as the most likely arena for the earliest phases of circumpolar globalization (Fig. 6). The extent to which the Greenland Norse voyaged to Baffin Island, up the Hudson Strait or deeper into Foxe Basin remains equivocal given adverse ocean currents and extensive sea ice during the main Norse sailing season, though hunting or trading possibly occurred at more accessible locations (see Supplementary Text). More generally, our results contribute fresh empirical insights to long-running debates about the likely location, timing, and motivations of early interaction between European Norse and Indigenous North American communities in the High Arctic. They confirm that elite consumption patterns in Europe fueled an insatiable demand for walrus ivory, and that provisioning these markets emerged as a major driving force that substantially shaped the trajectory of Greenland Norse interactions with Arctic Indigenous peoples.

Overall, our findings indicate that the major axis of walrus exploitation likely ran along a south-north “maritime corridor” linking Greenland Norse settlements to Northwest Greenland and into High Arctic Canada (Fig. 6). While all these conclusions remain tentative, they highlight the wider potential of integrating higher-resolution biomolecular sourcing methods with improved knowledge of Norse seafaring capabilities. Much larger assemblages of directly dated walrus artifacts should now be genetically sourced, and the emerging results may shift or further reinforce the preliminary interpretations presented here. Our study also highlights specific High Arctic regions requiring further archaeological fieldwork to better understand how different cultural groups operated and the extent to which they interacted. In particular, the traditional “Eurocentric” focus on Greenland Norse walrus exploitation should also be rebalanced with improved understanding of Tuniit and Thule Inuit mobility strategies, which may also have shifted over time as Greenland Norse hunting efforts and trading opportunities started to encroach (Fig. 6). Last, the methods used in this study highlight enormous potentials for a more comprehensive and truly circumpolar sourcing program to reconstruct the causes, conditions, and deeper ecological consequences of Arctic resource exploitation across different cultural and historical contexts.

## MATERIALS AND METHODS

### Materials: Sample provenance

See tables S1 and S2 for full details of sample provenance.

### Methods: Chronological inference

This paper reconstructs strategies of Greenland Norse (ca. 985 to 1450 CE) walrus exploitation to (i) understand which Arctic hunting grounds were used to supply ivory to markets and production centers in Europe, and (ii) determine whether these patterns changed over time. Resolving these questions requires working with three different kinds of chronological inference:

First, to source traded walrus artifacts back to specific Arctic hunting grounds, we needed to genetically match each “cultural” artifact back to the unique “biological” walrus stocks that had existed in specific locations during the period of Greenland Norse exploitation (ca. 950 to 1450 CE). While modern (or recent historical) biological samples can be used to reconstruct the modern genetic diversity of North Atlantic walrus stocks, the inherent risk is that current stocks and geographic distributions are a legacy of the more recent industrial-scale walrus exploitation. These devastating impacts and ongoing disturbances are likely to have led to the displacement, merging, separation, replacement, or extirpation of local walrus stocks, creating major uncertainties about the veracity of sourcing Greenland Norse artifacts on the basis of modern genetic diversity. To resolve these problems, we needed to reconstruct the contemporary genetic diversity and stock distributions during the period of Greenland Norse walrus exploitation. This required analysis of the ancient and historical mitogenome DNA of biological walrus samples (*n* = 100) obtained from a wide range of geographic locations, and also across relevant time periods, including areas where walrus stocks are known to have been extirpated by human pressures, including Iceland and the Canadian Maritimes (see table S1). To obtain these samples, we targeted archaeological contexts, sub-fossil geological finds, and other relevant collections. Samples are allocated to general chronological (or culture-historical) time periods, with specific dates provided where available. In this way, the precise calendar age of a particular walrus sample is less important; the main requirement was to target biological samples with sufficient chronological depth and appropriate geographic coverage. On the basis of these principles, our high-resolution phylogeography of walrus stocks (Fig. 1) reconstructs the genetic diversity and stock locations assumed to have existed at the time of Greenland Norse walrus exploitation (Fig. 2).

Second, we needed to genetically track the cultural artifacts back to these specific walrus stocks to understand Greenland Norse exploitation patterns, and whether these had changed over time (i.e., different hunting grounds used at different times). To resolve these questions, three chronological issues arise: (i) we needed to identify any likely time lags between walrus harvesting (in the Arctic) and the deposition of the cultural artifacts at trade and production sites (in Europe); (ii) to understand exploitation patterns over time, we needed to allocate each walrus artifact to general time periods in the history of the Greenland Norse; and, last, (iii) where possible, we needed to generate more specific age ranges for each artifact. We dealt with each of these issues in turn: (i) Identifying time lags (between hunting, shipment, and production). The Greenland Norse shipped “packages” of ivory back to Europe, with the tusks and teeth still attached to the front part of the skull (the rostrum). These packages were broken open at processing and production centers to extract the full length of ivory tusk, generating distinctive cultural waste that serves as a direct proxy for the wider ivory trade (*17*, *19*). We assume that processing (and discard of waste) occurred relatively soon after arrival (i.e., within years or a couple of decades after the hunt) because commercial value is added by converting the raw material into precious objects. In contrast, the valuable artifacts carved from the walrus ivory (e.g., items with religious significance or used for signaling social status) may have remained in circulation for generations (many decades or even centuries) before entering the archaeological record. Specifically, the 31 walrus artifacts [original data from Star *et al.* (*17*)] are described in table S2 and mainly consist of rostra production waste (*n* = 27), tusk fragments (*n* = 3), and a tooth (*n* = 1). Overall, 27 of 31 samples were production waste (rostra) reducing likely time lags between hunting, shipment, and processing. Most samples are from European trade or production centers (*n* = 27), and a few samples are from the Greenland Norse settlements (*n* = 4). (ii) Assigning artifacts to general time periods. The paper builds directly on previous research by Star *et al.* (*17*) and we use the same approach to chronological inference: (a) first, the walrus artifacts are dated by the archaeological context from which they were recovered (see details in table S2); this generated time bands of varying widths (Fig. 2); and (b) second, these data were used to allocate the walrus artifacts to two major historical periods in the Greenland Norse settlements: an Early Period and a Late Period (Fig. 2). These two periods are divided by the key date of 1120 CE, which marks the point at which Norse Greenland communities received their first bishop [i.e., early 1120s CE (*50*)], itself a reflection of the wider socio-political and economic transformations affecting Scandinavia and the North Atlantic (see the “culture-historical timeline” below). Assigning the walrus artifacts to these two broad chronological intervals enabled us to demonstrate that general patterns of walrus exploitation had shifted substantially over time (Fig. 2). (iii) Assigning specific ages to artifacts. Generating precise calendar dates for each of the 31 walrus artifacts is more challenging and was deemed beyond the scope of the current paper. The underlying problem was also highlighted by Star *et al.* (*17*). While C14 dating methods could be used to date the individual artifacts, this could only generate a radiocarbon age for each object. This age would then need to be calibrated to assign a calendar (historical) age, taking marine reservoir effects into account. These reservoir effects vary according to geographic location and other considerations and are a particular problem for walrus given its high fidelity to localized shallow-water feeding grounds (*10*). Without calculation of a precise local Δ*R* value to correct for all the potentially different marine reservoir effects across our wider study area, the direct dating of the samples would add further chronological uncertainty. Now that the walrus artifacts have been sourced back to more specific geographic regions, baseline data and proof-of-concept studies to support improved radiocarbon calibration can now begin and should be a future research priority.

Third, the sourcing results need to be embedded into a wider historical context to understand the causes, conditions, and consequences of Greenland Norse walrus exploitation. Key historical processes and transformations affecting walrus exploitation and the demand for ivory include (i) initial Norse expansion into the Northwest Atlantic (pre-1120 CE) and also (ii) the fundamentally different socio-political and economic dynamics that were emerging across Scandinavia and Europe during the Late Period of Norse Greenland, including the rise of various polities (ca. 1120 to 1450 CE). These wider historical transformations can be summarized as a culture-historical timeline (all dates in CE; for further discussion of Norse-Indigenous interactions, see Supplementary Text):

• 984 to 992: Erik the Red departs from Iceland and explores the west coast of Greenland (*51*), possibly traveling beyond Disko Bay (*52*).

• c. 985: Founding of the Greenland settlements (*51*). Leif Eirikson (born c. 970, died, c.1025) credited with bringing Christianity to the Norse Greenland settlements and being the first European to visit continental North America (*50*, *53*).

• 1021: dendrochronological date for timbers from the Norse settlement at L’Anse aux Meadows (Fig. 6) in the Canadian Maritimes (*54*).

• c. early 1120s: the Norse Greenland settlements receive their first bishop (*50*).

• Early 12th century: Ari Þorgilsson writes the *Book of the Icelanders*, the earliest example of the term “Skrælinga” (*55*).

• Late 12th century: the *Historia Norvegiae* mentions *Skraelings* living north of the main Greenland Norse settlements (*56*).

• After c. 1200: Weather and sea-ice conditions begin to worsen at the Western Settlement (*48*).

• Around c. 1250: Tuniit (Late Dorset) groups withdraw from High Arctic Greenland (*26*).

• 13th century: The Bear Trap storehouse (fig. S3) constructed on the western tip of the Nuussuaq Peninsula (*25*, *26*).

• 1250s: Novgorod begins to expand as a fur-trading power, becoming a direct competitor for Greenland Norse traders (*29*).

• 1250 to 1300: Norse runes carved at Kingitorsuaq, confirming expeditions and overwintering beyond Disko Bay (*25*, *31*).

• 13th century: Thule Inuit expansion from Alaska into the Eastern Arctic (*1*).

• After c. 1250 to 1350: Norse artifacts start to appear on Thule Inuit sites, particularly in Smith Sound, but also in the Canadian High Arctic (*9*, *24*).

• 1262 to 1263: Greenland and Iceland submit to King Hákon Hákonsson of Norway; beginning of embargo on all foreign trade north and west of Bergen (*29*).

• 1266 to 1267: Two Norse expeditions into the far north, described by a Greenland priest, possibly reaching Melville Bay (*6*, *51*).

• c. 1300: Peak of the Greenland Norse population at the Western Settlement (*57*).

• 14th century: Thule Inuit expansion southward along the west Greenland coast, with establishment of winter bases in the Disko Bay area (*26*).

• 1327: Peter’s Pence tax for Magnus Eiriksson’s crusade against Novgorod paid by the Greenland See, primarily via a large quantity of walrus ivory: Exact amount was unclear, but worth more than the annual tax from c. 4000 Icelandic farms (*5*, *25*, *29*).

• 1341: The Norwegian priest Ívar Bárðarson is sent to Greenland on behalf of the Bishop of Bergen, and reports that no Norse taxpayers are left at the Western Settlement (*52*).

• 1347: The *Skálholt Annal*’s entry for this year records a ship, with 17 men onboard, arriving in Iceland from Greenland, which had sailed to Markland; last known reference to the Americas before Columbus (*31*).

• 1350 to 1450: period of “exceptional climate instability” in Greenland (*58*).

• c. 1360: Ívar Bárðarson writes his description of Greenland, stating that the sailing route from Iceland to Greenland is no longer possible due to encroaching sea ice (*52*).

• 1379: The *Icelandic Annals* record that “the *skræling* attacked the Greenlanders and killed eighteen men and took two boys into slavery” [transl. A. Ogilvie (*49*)].

• c. 1380: peak of the Norse population at the Eastern Settlement (*57*).

• 1408: last written reference to the Norse occupation of Greenland (*59*).

• c. 1450: Eastern Settlement abandoned, end of Norse presence in Greenland (*57*).

To summarize, these different approaches to chronological inference enabled us to (i) reconstruct genetic diversity and walrus stock locations at the time of Greenland Norse exploitation (Fig. 1); (ii) use this phylogeographic analysis to source walrus artifacts back to specific Arctic hunting grounds; (iii) allocate traded walrus artifacts into two major historical periods to understand how walrus exploitation patterns shifted over time (Fig. 2); and (iv) use these results to better understand the emergent phenomenon of the “Arctic Ivory Road”—i.e., the evolving trade, interaction, and exchange networks that started to connect the Indigenous Arctic, Norse Greenland, the North Atlantic, and European urban centers via the commercial exploitation of natural resources located in the polar regions (Fig. 6). Further research can refine and develop these emerging insights.

### Methods: Using ancient DNA to reconstruct the genetic diversity and stock locations

As described above, accurate sourcing of walrus cultural artifacts required reconstruction of genetic diversity and stock locations during the period of the Norse Greenland settlements (Figs. 1 and 2). We targeted mitogenomes from biological samples (*n* = 100) to ensure sufficient geographic and chronological coverage (see table S1). All DNA work was conducted in dedicated laboratories at the Globe Institute, University of Copenhagen, following established aDNA protocols (*60*) as described in Ruiz-Puerta *et al.* (*10*). All raw DNA sequence data were mapped to a walrus reference mitogenome (NCBI accession: NC_004029.2) (*61*) using the PALEOMIX (v1.2.13.4) BAM pipeline (*62*), excluding the d-loop due to poor mapping. MapDamage (v2.0.9) (*63*) was used to assess the postmortem damage and confirm the authenticity of our ancient DNA. Adapters, ambiguous short sequences (<25), and low quality bases (*Q* ≤ 30) were removed with Adapter removal (v2.3.1) (*64*). Duplicates were removed with SAMtools (v1.3.1) (*65*) and MarkDuplicates (Broad Institute). Mitogenome haplotypes were called independently with ANGSD (v0.921) (*66*) using SAMtools and BAQ computation (*67*) against the reference walrus mitochondrial genome. Bases were not called for sites where depth of coverage was <3, and reads were removed if there were multiple best hits during mapping.

### Methods: Sourcing walrus artifacts to specific Arctic hunting grounds

The genomic sourcing of walrus artifacts is supported by phylogeographic analysis, in which the mitogenome “fingerprint” from a cultural walrus artifact is allocated to the biological phylogenetic clade of the walrus stock from which it was harvested (Figs. 1 and 2). This approach is made possible by the strong (maternal) population structure of walrus, with multiple discrete populations now identified in the North Atlantic (*10*, *68*), resulting in a well-resolved phylogenetic tree (i.e., there are several distinct local stocks, and each stock has a distinctive genetic identity). As discussed above, previous studies have used genetic methods to source ivory (*17*), but used a phylogeny built with short fragment mitochondrial DNA, rather than full mitogenomes, and used relatively modern Arctic reference samples that postdate industrial-scale walrus exploitation (*17*). Still, this pioneering study was able to define two large geographic walrus clades (western and eastern/mixed) and demonstrated that Norse walrus exploitation had shifted from direct hunting in Fennoscandian waters, followed by expansion of harvesting efforts into the Northwestern Atlantic in the early 12th century (*17*). However, more recent research, using mitogenome data, has indicated that the large “western” clade is, in fact, made up of several distinct walrus stocks, each located in different geographic areas, and, moreover, that a series of distinct stocks also existed during Greenland Norse walrus exploitation (*10*). This baseline work on genetic diversity establishes a much higher-resolution framework to track the cultural artifacts that passed through Norse Greenland back to more specific Arctic hunting grounds.

We built a high-resolution Bayesian phylogeny, using mitogenome data from biological samples sourced from different chronological periods and geographic locations (Fig. 1 and table S1) combined with mitogenome data from 31 walrus artifacts [data originally published by Star *et al.* (*17*)] into the Bayesian phylogenetic analysis (table S2). The Bayesian phylogenetic analysis was completed on all samples with at least 90% of breadth coverage using a relaxed clock model and 150 million iterations in BEAST 2 (v.2.5.1) (*69*), as described in Ruiz-Puerta *et al.* (*10*). The biological samples directly allowed us to define six stocks during the period of Greenland Norse walrus exploitation: an extinct stock from Iceland (II); East Greenland (III); West Greenland (IV); Northwest Greenland (North Water Polynya) (V); and Foxe Basin (I and VI). A further stock was identified in Svalbard (see pale blue shading in Fig. 1, stock not numbered in the current paper), plus an extinct stock in the Canadian Maritimes (see pale yellow shading in Fig. 1; stock not numbered in the current paper). Next, with every cultural artifact possessing a distinct genetic fingerprint, it was possible to genetically allocate each object to a specific biological walrus stock that had existed during the Norse presence in Greenland (Fig. 1). Chronologically, all sourced artifacts were allocated to either the Early Period or the Late Period of Norse Greenland [(*17*), see table S2], the results indicating that Norse harvesting strategies had likely evolved over time, with the North Water Polynya (stock V) becoming increasingly important (Fig. 2).

### Methods: Reconstructing Greenland Norse sailing vessels, routes, and journey times

To contextualize the results of the genetic sourcing, and further evaluate the veracity of the three different Norse exploitation scenarios, we used archaeological, historic, and ethnographic data to reconstruct two probable Greenland Norse vessel designs: (i) smaller boats with oars and sail, and (ii) larger expeditionary sailing ships (Figs. 3 and 4 and figs. S1 and S2). We (G.J.) also conducted experimental voyages in vessels directly comparable to those available to Greenland Norse communities, generating insights into sailing and rowing capabilities, plus estimations of likely cargo capacities. This enabled us to assess their relative voyaging capabilities and reconstruct possible sailing routes and journey times (Fig. 5 and table S3), drawing on paleoenvironmental evidence to establish robust comparisons between current conditions and those likely experienced by the Greenland Norse, particularly in relation to wind direction and sea-ice coverage (*70*–*73*). These combined insights enabled us to better understand Greenland Norse seafaring capabilities, including the different operating ranges of the smaller and larger vessels, as well as likely routes, possible anchorages, stopping points, and hunting grounds (Fig. 5). We concluded that Greenland Norse needed to choose between (i) voyages northwards from the main Norse settlements located in southwest Greenland, following the western coast of Greenland, as far north as the North Water Polynya: These expeditions were risky, but still feasible within one summer sailing season, but only with the larger expeditionary sailing vessels that were owned by wealthier farmers and social elites; and (ii) westward expeditions over to Baffin Island, Labrador, and deeper into Foxe basin, which we concluded were less likely given lingering sea ice and difficult sailing conditions; voyages in this direction would also have required at least one overwintering, even with the larger sailing ships. While earlier exploration voyages may have taken these risks into consideration, more routine walrus harvesting expeditions appear to have targeted the North Water Polynya as the more viable option for the small Greenland Norse communities. For additional information on Greenland Norse seafaring capabilities, plus interactions with Arctic Indigenous peoples, see Supplementary Text.

## Supplementary Materials

### The PDF file includes:

Supplementary Text Figs. S1 to S3 Table S3 Legends for tables S1 and S2 References
