# Supplementary material for: Greenland Norse walrus exploitation deep into the Arctic
Source: Sci Adv. 2024 Sep 27;10(39):eadq4127. doi: 10.1126/sciadv.adq4127 (PMC11430631; doi:10.1126/sciadv.adq4127)
Supplement: Supplementary file 1 — Supplementary Text Figs. S1 to S3 Table S3 Legends for tables S1 and S2 References [file sciadv.adq4127_sm.pdf]

Science Advances
AAAS

# Supplementary Materials for

## Greenland Norse walrus exploitation deep into the Arctic

Emily J. Ruiz-Puerta *et al.*

Corresponding author: Emily J. Ruiz-Puerta, emily.puerta@sund.ku.dk; Greer Jarrett, greer.jarrett@ark.lu.se; Morten Tange Olsen, morten.olsen@sund.ku.dk; Peter D. Jordan, peter.jordan@ark.lu.se

*Sci. Adv.* **10**, eadq4127 (2024)
DOI: 10.1126/sciadv.adq4127

**The PDF file includes:**

Supplementary Text
Figs. S1 to S3
Table S3
Legends for tables S1 and S2
References

**Other Supplementary Material for this manuscript includes the following:**

Tables S1 and S2

## SUPPLEMENTARY TEXT

### Experimental and historic insights into Norse sailing and inter-cultural contacts

*General environmental conditions*

The west coast of Greenland is today characterised by prevailing northerly winds, unstable summer weather with frequent storms, rain and fog, and major inter-annual variations in the extent, location, and concentration of sea ice (*74*), This means sailing is inherently risky because weather conditions and sea ice coverage can change drastically in a matter of hours (74). The complex coastal topography between the interior Greenland ice sheets and surrounding ocean can generate strong katabatic winds with sudden, powerful gusts, particularly through the inner fjords, creating major risks for sailing vessels (*70*). Documentary and paleoenvironmental evidence from across southwestern Greenland indicates that similar meteorological and environmental conditions to those observed over the last century probably existed in the Early Period of Norse Greenland settlements, albeit with higher frequency of southerly and katabatic winds, and reduced summer fog (*70–73*). However, conditions began to worsen around the Western Settlement already in the mid-13$^{th}$ century, with increased storms and greater sea ice coverage. These changes began to impact the Eastern Settlement in the mid-14$^{th}$ century, with contemporary sources noting increased sea ice and a consequent decline in trading voyages from Scandinavia and Iceland to Greenland (*29*, *48*, *58*).

Despite high variability in the maritime climate of western Greenland, some general seasonal patterns observable today would also have impacted upon the sailing options of the Greenland Norse. The extent of the sea ice varies significantly from year by year, but ice-free conditions can generally be expected between July and October as far north as Upernavik (*74*, *75*). However, sailing beyond this point often proves impossible until July or even August, adding uncertainty and narrowing the time window for longer-range expeditions seeking to return within one summer season. Likewise, sea ice generally reaches its minimum extent in late September, but then forms rapidly again after this point, adding further constraints and uncertainty (ibid). Ocean circulation also impacts conditions with the north-flowing West Greenland Current keeping the Greenland side of the Davis Strait ice-free for longer periods, while conditions are more challenging along the coast of Baffin Island and Labrador, where access to the coast is generally blocked by dense pack-ice until August (*31*). To summarize,

the most predictable sailing conditions and safest seascapes are to be expected along Greenland's west coast from the Norse settlements through to the North Water Polynya; voyages to Baffin Island, and across to Labrador and into Foxe Basin are fraught with greater risk and uncertainty, especially in relation to the sea ice (Fig. 5, Fig. 6).

*Defining the length and timing of Greenland Norse sailing season*

These combined climatic and environmental parameters define a sharply constrained annual sailing season for Greenland Norse walrus harvesting operations. More distant walrus stocks could have been reached, but at increased risk of getting stranded, requiring overwintering away from home. Norse sailing decisions and strategies also need to be understood in the context of the socio-economic and demographic characteristics of the main Greenland Norse settlements, which were situated at the remote outer edges of the European cultural world. Previous research has identified the Western Settlement as the main community involved in the *Norðrsetur* hunt (*6*, *29*, *51*). For this community, the early summer was taken up by the hunting of migratory harp seals, which passed the outer coast in May and early June, generating wild food supplies during the leanest and hungriest months at the end of the long winters (*49*).

Northbound walrus hunting expeditions could have departed immediately after this task. However, the crews would be needed back at the farmsteads by late August to take part in haymaking which created fodder for livestock over the winter. These two vital tasks – seal hunts and haymaking - define a narrow window of opportunity for the *Norðrsetur* hunt, which probably lasted around two and a half months, starting early June and ending late August (*6*, *25*). Unfortunately, the opening of this travel window aligns particularly poorly with the lingering sea ice along the west coast of Baffin Bay and the Davis Strait, meaning that Norse voyages to Baffin Island and Labrador were unlikely to have been undertaken from the Western Settlement on a regular annual basis, unless possible overwintering was factored into the plans: crews would then be absent for both haymaking and also the Spring seal hunt. These domestic task-scheduling conflicts would have added further risks and uncertainties to the already hazardous voyages to the west, especially if crews decided to venture deeper into Foxe Basin (Fig. 5, Fig. 6).

*Reconstructing the size and diversity of Norse Greenland vessels*

Our research indicates that (a) Norse voyages to the northern hunting grounds were conducted in two different kinds of vessel (compare Fig. 1 and Fig. 2, plus fig. S1 and fig. S2), and that (b), these had distinctly different operating ranges, which would have structured opportunities for walrus exploitation, and for the Greenland Norse social groups and wider networks that organised, participated and profited from the harvesting operations. No complete examples of Greenland Norse vessels have survived, but the boats and ships used for the *Norðrsetur* hunt can be reconstructed from a range of sources, including fragmentary finds, historical records, historical analogues and archaeological remains of boathouses. The direct archaeological evidence for Greenland Norse vessels is restricted to hull fragments recovered from Norse settlements and some Thule Inuit sites (possibly parts scavenged from wrecked ships) but these are too small to enable vessel sizes and cargo-capacities to be reconstructed. One small model of a four-oared boat has been recovered from Umîviarssuk in the Western Settlement. Overall, these combined insights suggest that the vessels of the Greenland Norse were probably single-masted, square-rigged rowing and sailing boats of the wider Nordic clinker tradition (*24*, *31*, *51*, *76*, *77*):

- **Smaller boats:** Written sources confirm that the most common type of vessel used by Greenland Norse was the *sexæring*, a relatively small boat of around 7 - 10 m length with six oars and a single square sail, probably owned and used by individual households (*25*, *52*). These small boats are thought to be analogous to the clinker-built fishing boats of Shetland and western Norway, which bear variants of the same name, and were in use until the early 20$^{th}$ century: the sixareen (Shetland) or *seksring* (Norway) (*42*, *43*, *78*) (Fig. 3, fig. S1).
- **Larger "expeditionary" sailing ships:** A larger class of Greenland Norse vessel, referred to as *skútur* or *ferja* in the sources, was probably constructed and operated under the direction of richer farmers and elites, though there is some ambiguity about their precise size and cargo-capacity (Fig. 4, fig. S2).

Vessels of this larger class were used widely by the Norse for general inshore travel, longer-range expeditions between Iceland and Norse Greenland, and exploration voyages to the Canadian Maritimes (*6*, *52*). The 14$^{th}$ century *Skálholt Annal* states that in 1347 (CE), there "came a ship from Greenland, smaller in size than the small vessels that trade with Iceland. […]. There were seventeen men onboard, and they had sailed to Markland" (probably coastal

Labrador, see Fig. 4) (*31*). This indicates that the ships of the Greenland Norse were smaller than the Norwegian ships that traded with Iceland, which were reaching lengths of 25-30 m by this date (*79*). To have reached Markland, this particular vessel must have been small enough to manoeuvre through the iceberg-filled waters, yet capacious enough to accommodate a crew of 17 persons. Remains of boathouses have also been identified at the Eastern Settlement and suggest vessel lengths of up to 18-20 m (*80*, *81*). Together, this evidence suggests that Greenland Norse were operating a class of larger "expeditionary" ships of 16-20 m in length, which were substantially larger than the small rowing boats described above, but perhaps somewhat more compact than the medieval Scandinavian ocean-going cargo ships. Keller (*29*) and Nedkvitne (*6*) have suggested that the 11th century *Skuldelev 3* wreck could have been similar to some of the larger class of vessels employed in the *Norðrsetur* hunts. This particular boat was built in Denmark and originally measured 14 m in length, putting it below our estimates for the larger Greenland Norse vessels; the original rig and sail plan also remain unknown, as these elements were not preserved. Alternative, and more well-documented analogues for the larger Greenland Norse boats may include the Norwegian *fembøring* or *Lofotbåt* and the *vaterbords-jekt*, which have similar dimensions of around 14 – 20 m, and were used for the stockfish trade into the late 19th century (*42*, *82*). Given the acute shortages of iron and boatbuilding timber endured by the Greenland Norse, combined with the small populations at the main settlements, we can potentially envisage *Norðrsetur* hunts involving 10-15 vessels during the peak of the Western Settlement's population (*25*, *57*), with the majority consisting of the smaller six-oared boats built, owned and crewed by local farmers, along with a handful of the larger ships likely sponsored by wealthier farmers and social elites.

*Reconstructing performance characteristics of different Greenland Norse vessels*

It is also important to understand the specific performance characteristics of the two different classes of vessel, given sailing conditions, the length of the summer voyaging season, and the different hunting grounds that were being targeted by the Greenland Norse in different historical periods (Fig. 2). One key issue is whether both classes of vessel were able to reach the distant walrus hunting grounds in the far north. The closest analogy to the smaller six-oared Norse boat is probably the Shetland sixareen; its length-beam ratio is closer to that of the Umîviarssuk model than that of the sleeker Norwegian *seksring*, giving it more space to carry bulky cargo. This is an important consideration given that Greenland Norse transported ivory tusks that were still attached to the rostrum, as well as bulky walrus hides (*6*). Sixareens were

usually 7.5-9 m in length and crewed by 6-7 people. While these are profoundly seaworthy craft, extremely well-adapted to the large swell and gusty winds of their home regions (*42*, *43*, *78*), they are not suited for long overnight passages as they provide no shelter from the elements and cannot accommodate for both cargo and a sleeping, off-duty watch. We envisage that on summer hunting expeditions, these small boats would have been rowed in 12 hour stints, with the crews resting up on land between these stages (*6*). In contrast, the larger sailing vessels could be sailed non-stop under favourable conditions with the larger crews working and resting in different watches, saving time, and enabling substantially longer voyages within the time window (*83*).

Much of the literature on the *Norðrsetur* hunt has been founded upon an ambiguous passage in a 17th century document known as *Grœnlands annál*, particularly from the copy made by Björn Jónsson of Skarðsá (d. 1655), probably in 1643, known as *AM 115 8vo(A)*, and reproduced in Halldórsson (*52*). The text reports a series of distances between named locations along the west coast of Greenland, which are listed in terms of the number of rowing days between them. The original information is derived from an older booklet which has since been lost (*gömlu kveri*). The passage begins with a list of the fjords and churches of the Eastern Settlement, and then continues northwards along the coast:

"Þá eru Miðfirðir næst *byggðir; þá heitir *enn einn Kolluf(jörður), annar Dýraf(jörður), þá Þórvaldsfjörður, Steinsf(jörður), Bergþórsf(jörður). Þá er vj daga róður | vj mönnum til Vestribggðar sexæringi (þá telur þar upp firði). Þá er ú hinni vestri byggð til Lýsufjarðar vj daga róður; þaðan sex daga róður til Karlbúða, þá iij daga róður til Bjarneyjar, xij daga róður umhverfis – ey Eisunes; Æðanes fyrir norðan."

This translates as:

*Then the Middle Settlement is the next settlement; one is called Kollufjörður, another Dýrafjörður, then Þórvaldsfjörður, Steinsfjörður, Bergþórsfjörður. Then it is six days of rowing [for] six men to the Western Settlement [in] a six-oared boat (then count up the fjords). Then from the Western Settlement to Lýsufjarðar there is six days of rowing; from there six days rowing to Karlbúða, then three days rowing to Disko Island, twelve days rowing around -- island Eisunes; Æðanes is to the north.*

(translation by Sigurður Snæbjörn Stefánsson, January 2024).

Halldórsson believed this passage had been somewhat corrupted, and suggested several corrections (*52*). Other authors have offered differing interpretations of this passage (*6*, *31*). Table S3 lists the locations mentioned in this passage and their possible distances from the Western Settlement according to a literal reading of the text, as well as the various interpretations offered by recent researchers (*6*, *31*, *52*). In our analysis, we have assumed that "the Western Settlement" listed in *Grœnlands annál* refers to the entry point to the deeper fjord system in which this Norse settlement was located, and not a particular farm or hamlet within this inner chain of farmsteads (the text is explicit that rowing times within the fjord are not included). It seems likely that the larger Greenland Norse ships, which were designed for sailing but were difficult to row, would have been moored at locations near the outer coast. This means that long-range sailing distances along the coast should be calculated from the vicinity of modern-day Nuuk (*83*–*85*).

As outlined in table S3, the different interpretations of the original passage generate quite substantial differences in the rowing speeds required to cover the distances between the named locations. To evaluate these interpretations, we made use of experimental trials conducted on comparable vessels in Scandinavian waters (Fig. 2). These trials demonstrated that a *fyring*, a traditional Norwegian boat of slightly larger size than the *seksring*, could be rowed for several hours by an amateur crew of 7 (with approximately 250 kg of ballast, 6 rowers and one person steering), achieving an average speed of 1.8 knots; however, the crew could not maintain this tempo indefinitely and would need to rest at nighttime. This estimated speed aligns well with the interpretation presented in the the *Grœnlands Annal*, which states that it took 15 rowing days to reach Disko Island in a small boat from the Western Settlement. If we assume that the start and end points are Nuuk and Qeqertarsuaq, that equates to 345 nautical miles, which would mean rowing 23 nautical miles per day over 15 rowing days, requiring an average Speed over Ground of 1.9 knots, a respectable pace considering the prevailing headwind.

In contrast, Ljungqvist (*31*) has calculated that the same journey could have been sailed within only 4 days using one of the larger class of Greenland Norse sailing vessels (Fig. 3), which would 'save' 11 days on the outward journey alone. This estimate assumes non-stop sailing, and equates to 86,25 nautical miles per 24 hours, which is well below averages for Norse ocean-going vessels commonly reported in contemporary written sources (*84*, *86*). However,

it aligns well with the daily averages obtained from experimental trials with a Norwegian *fembøring* (Fig. 4), and with the estimate of 80 nautical miles per 24 hours reported by Wallace (*87*), based on the crossing of the Davis Strait in 1998 by *Snorri*, a reconstruction of the 11th century *Skuldelev 1* wreck (*83*). Given the adverse and unpredictable sailing conditions along the western Greenland coast, we employ this more conservative estimate in our analysis.

We also estimated likely cargo capacities of the two kinds of Greenland Norse boats, using experimental reconstructions and ethnographic parallels: (a) the smaller six-oared boats can be compared with the Shetland sixareen, which can transport approximately 1 ton of cargo in addition to a crew of 6-7 (*31*, *43*, *78*); (b) the precise capacity of the larger Greenland Norse ships is less certain, given ambiguity over their precise dimensions: the *Skuldelev 3* vessel had a cargo capacity of only 4.5-5 tons (*29*), while the *Skuldelev 1* could ship up to 20 tons of cargo (*42*), which we take to be near the upper limit for the larger class of Norse boats involved in the *Norðrsetur* hunt.

*Norse walrus hunting strategies*

The *Norðrsetur* hunt would also have been influenced by the behaviour and mobility of walrus. These animals typically feed on marine bivalves at shallow depths of <100 m and then occupy haul-out sites on land or sea ice. McGovern (*25*) used bathymetric and marine sedimentary records to identify a series of likely feeding and haul-out areas along the west coast of Greenland. Zooarchaeological evidence from the Greenland Norse settlements indicates a distinctive walrus butchery pattern, with animals skinned at kill sites to remove the hide, and the skull chopped into sections to remove the valuable ivory tusks, which were left attached to the maxillae (*25*). There is no evidence that Norse hunters developed toggling harpoons nor adopted them from the Thule Inuit, so walrus were unlikely to have been hunted in the open water. Instead, they were probably harvested at haul-out sites, driven into the shallow surf from the sea, corralled on beaches by hunting dogs, and then finished off with lances. Similar strategies are reported from Svalbard and Sable Island in the 17th and 18th centuries, enabling a group of hunters to kill several hundred animals per day (*39–41*).

The hide and *rostrum* of a large adult walrus weigh approximately 52.5 kg (*25*). This means that one six-oared boat with a cargo capacity of 1 ton could have shipped 19 sets of hides and maxillae (even more if the bulky hides were discarded in favour of extra ivory "packages"). In

contrast, a larger Greenland Norse sailing vessel could transport between 85-380 sets (assuming a cargo capacity range of 4.5-20 tons). Assuming that Greenland Norse crews were able to operate as effectively as hunters in Svalbard and Sable Island, then the 85-380 walrus kills required to fill one of the larger Norse cargo ships could easily be culled, processed and loaded onto ships within 1-2 weeks, depending on hunting conditions, the size of the crew and the capacity of their vessel. The quantity of ivory paid by Greenland Norse as a crusade tithe in 1327 has been estimated at 190-260 pairs of tusks, which would equate to only 1-3 of the larger expeditionary ships returning from the hunt fully laden with walrus products (*5*, *25*, *29*).

Norse mobility strategies during these hunts are not clear, but they may have operated from central base camps, and then journeyed out to target specific haul-out sites, returning with hides and processed skulls. Secure storage facilities to protect the contents against roaming polar bears may also have been part of this strategy. One likely example is the Bear Trap (fig. S3), a probable Norse storage facility on the northwestern tip of the Nuussuaq Peninsula (Fig. 5 (*25*, *26*)). To summarize, the culling and processing operations at remote hunting grounds could have been completed quite quickly, within a couple of weeks at most (6). This insight means that voyaging times were a more important factor in determining which class of vessel could reach the different walrus hunting grounds.

*Estimating Norse journey times to different Arctic hunting grounds*

We calculated journey times to the different Arctic hunting grounds, taking into consideration the performance characteristics of the two different vessel types. These estimations are based on experimental sea trials conducted with traditional Norwegian vessels in Scandinavian waters, and could be further refined by undertaking voyages along the west coast of Greenland. First, it is assumed that Norse hunters had approximately two and half months to reach and return from any of the hunting grounds. Around half of this time would probably have been spent weathering storms on land and waiting for favourable winds, leaving only 30 days of actual journey time once hunting and processing time is accounted for. Second, at 23 nautical miles per day, the six-oared boats could cover 690 nautical miles in 30 days, or 345 nautical miles in 15 days in each direction. This makes it possible to reach Disko Bay rowing from the Western Settlement, but not further. Importantly, this logistical limit on the operating range of the smaller boats would have been further enforced by the exposed and unpredictable conditions north of the Nuussuaq Peninsula (*75*). Third, we estimate that the larger Norse ships

could be expected to travel c.80 nautical miles per day, covering 2,400 nautical miles in 30 days, meaning a maximum range of 1,200 nautical miles in 15 days in each direction (roughly the same distance as sailing from the head of the Oslo Fjord to the north coast of Spain). Crucially, this would place crews from the Western Settlement within potential voyaging distance of Pikialasorsuaq (North Water Polynya), and perhaps even the Kane Basin, but probably only on years with exceptionally favourable sailing conditions.

The superior operating range of the larger sailing ships is hinted at in the *Grœnlands annál*, which states that "The Greenlanders sail towards the north to the wilderness beyond the uninhabited lands, or the peninsula, both for wood and fishing" (*52*: translation by Sigurður Snæbjörn Stefánsson). The term used in Icelandic is *siglingar*, which in other contemporary sources such as *Landnámabók* is assumed to refer to open-sea voyages rather than coastal navigation. It is possible that the "uninhabited lands, or peninsula" refer to the area usually exploited by Norse hunters, i.e. the coast as far as the Nuusuaq Peninsula, while the "wilderness beyond" refers to Pikialasorsuaq (North Water Polynya), which could only be reached by crossing the open ocean west of Melville Bay. More favourable conditions on the voyage south (with prevailing northerly winds and less sea ice coverage in the late summer) would have made these return journeys somewhat faster than the outward trips.

To summarize, we conclude that the increasing reliance on the North Water Polynya in the Late Period of Norse Greenland required the use of the larger 'expeditionary' vessels, which were only owned by the wealthier farmers and members of the elite. These social factors mean that only the closer walrus hunting grounds would have been accessible to poorer households working with smaller, six-oared boats.

*Locating Norse stopping points along the west coast of Greenland*

To better understand the northward hunting expeditions that departed from the Western Settlement towards the North Water Polynya (Fig. 5), we examined evidence for possible anchorages and stopping points along the west coast of Greenland. Access to such locations would have improved the feasibility of journeys, offering shelter, supplies, and opportunities for emergency overwintering, and would therefore increase the probability of Norse presence at the North Water Polynya. There are indications that natural harbours were used to establish storage facilities and hunting stations. Several factors may have influenced the choice of

location, including shelter from storms and northerly winds, proximity to the outer coast (providing access to the open sea, and reducing the danger of katabatic winds and becoming trapped by pack ice), as week as access to walrus haul-out sites near to shallow water feeding banks (*13*, *25*).

The Bear Trap site mentioned above fulfils all these criteria (fig. S3) (*26*, *88*). This location at the northwest tip of the Nuussuaq Peninsula remains ice free for most of the year, and offers a protected harbour during bad weather, a site function that persisted into recent times (*17*). Other hunting stations are mentioned in the written sources and ethnographic records, but their locations, infrastructure and precise role in walrus harvesting operations need to be better investigated archaeologically (*25*, *52*), though they probably shared similar affordances to the Bear Trap site (25, 91). Running from south to north, similar stopping and harvesting points may have been located at regular intervals along the western coast of Greenland, perhaps separated by two-days sailing time (ca. 160 nautical miles). Within this interval, it is possible to anticipate likely weather conditions, informing decisions about continuing with the voyage or waiting out unfavourable weather.

Karlbúða is one example of a way-station mentioned in the written sources (25,26). Using information from table S3, it is possible to estimate that it was located around 6 to 12 rowing days north of the Western Settlement, with three further days of rowing before reaching Disko Island. If boats travelled 23 nautical miles a day, this would place this stopping point around 138 to 276 nautical miles north of modern-day Nuuk, and around 69 nautical miles south of Disko Island. We suggest that according to the additional criteria established above, Karlbúða was probably located in a south-facing bay between 67° 50' N - 68° 10' N, in the vicinity of modern-day Attu (c.f. 25,26). This area offers good access to walrus feeding banks as well as shelter at the end or beginning of a long stretch of exposed coastline. Following the same criteria, another possible way-station may have been located at the mouth of the Kangerlussuaq Fjord, which sits midway between Nuuk and Attu (*25*).

Located 140 nautical miles further north than the Bear Trap is the island of Kingittorsuaq, where a Norse runestone was discovered (Fig. 5). It was carved sometime between 1250-1300 CE (*25*, *31*), which would place it in the Late Period of Norse Greenland, when more ivory was being harvested from the North Water Polynya (Fig. 2). The likely month of carving, estimated as April or May, is also significant, as sea ice would still have blocked the coast,

indicating that the crew had probably overwintered in this area, perhaps after being stranded on the journey home. Further north, Seaver (*30*) presents ethnographic accounts of a Norse settlement in the Upernavik area, which we tentatively locate in the relatively protected archipelago between 72° 40' N - 73° 20' N. This may represent a fourth waystation on the *Norðrsetur* route (Fig. 5). From here, Norse sailors may have headed towards Kitsissorsuit (Edderfugleøerne) before attempting to cross Qimusseriarsuaq (Melville Bay). The *Greenland Pilot* (*75*) reports that whaling crews used the topographic heights of these islands to inspect the sea ice conditions in the waters further north, a practice that may also have been common in the Greenland Norse period. From Kitsissorsuit, Melville Bay could be crossed in two days.

Another day's sailing from here would place Norse crews in optimal walrus harvesting territory, and also in close proximity to Uummannaq and Nuuliit, two of the more southern Thule Inuit settlements near the North Water Polynya (*76*, *89*; Fig. 5). Together, this chain of Norse waystations created a structured, safe and familiar sailing route that ran from the Western Settlement up to Pikialasorsuaq (North Water Polynya) and perhaps even to the Kane Basin, with sheltered anchorages and stopping points located 2-3 day's sailing distance from each other. Operating along this familiar 'maritime corridor' would have made long-range harvesting expeditions more feasible. Such a chain of havens is similar to the more formalised system of inns and stopping points along the Norwegian coast established by royal decree in the 13th and 14th centuries (*84*).

To summarize, available evidence indicates that the Greenland Norse made preferential use of a familiar coastal route that led from their main settlements up to the North Water Polynya, which came to dominate walrus exploitation in the Late Period (Fig. 3).

*Locating Norse stopping points: Baffin Island, Labrador, entrance to Foxe Basin*

We also considered possible westward expeditions across the Davis Strait to Baffin Island, into the Hudson Strait, and to Labrador. Access to all these areas would have been severely impeded by lingering sea ice well into the summer sailing season. Voyages beyond this area, and deeper into Foxe Basin, seem even less likely due to the adverse currents in the Hudson Strait. However, documentary and archaeological evidence confirms that the Norse were visiting what is now the Canadian coastline (i.e. Helluland and Markland, see Fig. 6). For example, the *Skálholt Annal* (see above) describes the arrival of a ship in Iceland in 1347 CE that had visited

Markland, a destination that did not appear to have been exceptional; Greenland Norse may thus have undertaken occasional visits to the outer coasts of North America, probably to harvest timber for boat building and construction, which would have required larger vessels to transport the timber back to the main Greenland Norse settlements. Substantial forests only start along the Labrador coast south of latitude 58° N, in the vicinity of Napartok Bay (*31*). A direct crossing from either of the Greenland Norse settlements would be a risky endeavour, as Martin Frobisher found during his expeditions in the 1570s (*90*).

It is at least equally likely that Greenland Norse expeditions crossed the Davis Strait further north, and then followed the Labrador coast southwards, as seems to be described in the *Saga of Erik the Red* (*85*). In this case, the Greenland Norse would likely have made use of familiar waystations similar to those running along the western coast of Greenland, but further archaeological fieldwork is needed to confirm this possibility. The main open sea crossings on this route are the Cumberland Sound and the Hudson Strait; anchorages on both sides of these landforms may have been frequented by Norse sailors on their way to Markland, leading to possible encounters with Tuniit communities, and later, with the expanding Thule Inuit populations.

Interestingly, a separate walrus stock also existed in the Canadian Maritimes but has since been extirpated. Our sourcing work indicates that no ivory was being harvested by Greenland Norse from this stock (see main text, table S1, table S2), which suggests that Norse exploration voyages to this area, and the establishment of the L'Anse aux Meadows site, were motivated by other factors. These may have included information gathering, timber harvesting, and searching for suitable land for permanent settlement. In contrast, the *Norðrsetur* expeditions to northern Greenland were focused entirely on acquiring ivory and then shipping this valuable commodity back to European markets.

To summarize, Greenland Norse voyages westwards, to coastal North America, were hazardous operations, requiring at least one overwintering and possibly more, and may have had other goals, including the harvesting of timber.

*Summary: Greenland Norse seafaring capabilities*

We conclude that:

- Greenland Norse vessels visited Baffin Island, Labrador, and Newfoundland but were primarily involved in exploration and timber harvesting. The extreme distances and uncertainties involved in these expeditions would probably make them multi-year endeavours, with overwintering on the Labrador and Newfoundland coasts. Such expeditions would have been analogous to trading journeys to Iceland, which were also undertaken over two summers (86).
- In contrast, the 'maritime corridor' running along the west coast of Greenland, which linked their main settlements with the High Arctic, seems to have been used on a seasonal and more regular basis (Fig. 5).
- This 'maritime corridor' can be broken down into two distinct sections: (a) a more general marine thoroughfare that ran from the Eastern Settlement, via the Western Settlement, and as far north as the Nuussuaq Peninsula. This route was navigable by both the small and larger vessels and formed the initial focus of walrus harvesting in the Early Period, primarily involving direct Norse hunting; (b) the High Arctic expeditions that focused on the ecological 'hotspot' of the North Water Polynya, which were beyond the reach of the smaller boats and required larger 'expeditionary' sailing ships, which could only reach this area in years with more favourable conditions.
- The vital importance of the larger sailing ships in the Late Period of Norse Greenland is significant because these were owned by richer farmers and social elites (and not poorer households). By this point, the Greenland Norse settlements had submitted to the Norwegian crown, diverting the lucrative North Atlantic trade networks through Bergen (29). This would have created a very different socio-political context for the organisation of ivory harvesting at ever more remote Arctic hunting grounds. Possibly, these longer-range efforts may have fallen under the control of powerful individuals and elite networks. This pattern would contrast with the more opportunistic and individually fuelled acquisition strategies that likely defined the Early Period of Norse Greenland, when use of small boats by independent householders could also generate income and personal status through harvesting the accessible local stock.

*Inter-cultural encounters and exchange: Greenland Norse, Tuniit, Thule Inuit*

Our sourcing work confirms progressive geographic expansion in the range of Greenland Norse harvesting activities (see main text). One corollary to this conclusion is the increased likelihood of encounters and then more formalised exchange relations with the Tuniit and especially the Thule Inuit (Scenario 3). Here we explore the available evidence, bearing in mind the wider culture-historical processes underway, which saw the Tuniit widely present when the Greenland Norse settlements were established, and then the displacement of these groups by expanding Thule Inuit, especially after 1200 CE. After this, the Thule Inuit continued to expand southwards into Greenland, eventually replacing the Norse population in Greenland after their settlements were abandoned (1). Evidence for the earliest 'great circle' European Norse and Indigenous North American encounters takes two forms: written records (from a Norse perspective) and archaeological data. Ari Þorgilsson's *Book of the Icelanders* (*Íslendingabók*), likely written in the early 12th century CE, is widely believed to be the first source to describe "Skrælinga"—a generic term used to denote Indigenous groups encountered in Greenland and Canada. The term appears again in the *Historia Norwegie,* probably composed in the second half of the 12$^{th}$ century CE, to refer to people living "north beyond the Greenlanders" (*56*).

- Norse-Tuniit (Late Dorset Pre-Inuit) Interactions: These early written references almost certainly refer to the Tuniit, as Thule Inuit did not arrive in High Arctic Greenland until after these documents were written. The archaeological evidence for direct Norse-Tuniit encounters is more equivocal, though there would have been around three centuries of temporal overlap, but with groups largely focusing on different geographic areas. Possible contacts have been inferred through objects recovered from Tunnit sites that are attributed to (or perhaps inspired by) interactions and cultural exchange with Norse visitors. Examples include spun cordage and Norse-style whetstones recovered from four Tuniit sites located in northern and southern Baffin Island, and in northern Labrador (*91*), plus traces of a copper-tin alloy within a stone vessel, which may indicate European-style smelting (*92*). However, the case for Norse-Tuniit contacts is undermined by the fact that radiocarbon dates appear to place these 'European' objects into earlier phases of the Tuniit (Dorset) culture, which would pre-date the widely accepted onset of the Greenland Norse presence, though this point is contested (*91*). As noted in the main text, more compelling evidence comes from the Smith Sound region, located between Northwest Greenland and Ellesmere Island, where a fragment of a smelted brass pot was recovered from a reliably-dated Tuniit context (*26*). We conclude

that some initial encounters between Tuniit and Norse must have taken place, and that these related to Greenland Norse exploration of the wider region, with meetings most likely taking place in Northwest Greenland (Smith Sound) and on Baffin Island, and possibly in coastal Labrador.

- Norse-Thule Inuit Contacts: There is more substantial archaeological evidence for interactions between these groups, mostly in the form of Norse artefacts recovered from Thule Inuit sites across Western Greenland (*26*, *93*) and deep into Arctic Canada (*47*, *94*, *95*), though some items could have been scavenged from shipwrecks (76). Our insights into ivory harvesting at the North Water Polynya add an important new line of evidence to these debates. Higher levels of interaction are understandable given the presence of both groups around the North Water Polynya. Moreover, in the closing decades of the Late Period of Norse Greenland, the Thule Inuit were also expanding down the western Greenland coast, possibly due to climatic deteriorations in the High Arctic, with both groups briefly operating in and around Disko bay (26), and contemporaneous occupations reported as far south as Sandhavn (*27*). We conclude that more formalized exchange relations may have started to form around the North Water Polynya in the Late Period, possibly with Thule Inuit initially gifting ivory (and possibly Arctic furs), and perhaps later engaging in more opportunistic bartering for Norse goods and equipment. In understanding these cultural encounters, it is important to bear in mind the different social norms and expectations of the participating groups, but also how these may have evolved over time as interactions became more structured and predictable. Norse interests were clearly focused on walrus ivory, but what the Thule Inuit could receive in return has remained ambiguous. Interpretations often assume the value of iron to Thule Inuit, but the Norse Greenland settlements were also short of this vital resource. The role of Norse elites in organising the larger expeditionary vessels may be significant here, as iron could be accumulated prior to the voyages and then traded in the High Arctic. Overall, however, the volume and character of Norse goods entering Thule Inuit contexts appears to be limited and rather eclectic, and more archaeological research is needed in likely interaction areas, including the North Water Polynya. Despite this ambiguity, the growing evidence for substantial Thule Inuit interactions with the Greenland Norse are compelling, with European urban demand for walrus ivory 'pushing' Norse expeditions deeper into the High Arctic. We also need to calibrate our underlying expectations of the archaeological record in these polar regions; very limited volumes of European trade

goods have been recovered from Greenland Inuit sites that date to the 18th C CE, when extensive inter-cultural interactions and formalized exchange relations are well-documented in the historical records (95). Finally, the other enduring puzzle is why the Greenland Norse appear to have been persistently disinterested in acquiring other potentially useful technologies, equipment and practices from the Arctic-adapted Thule Inuit, including toggling harpoons, skin-boats and insulated winter clothing made from skins and hides. This enigmatic combination of High Arctic interaction and cultural separation suggests that while two very different social worlds converged via shared interests in walrus exploitation, the deeper cultural consequences of these early encounters remained quite limited, at least during the earliest phases of circumpolar globalization. By the close of the 15th C CE, the Norse had departed and the Thule Inuit were expanding into southern Greenland. More intensified processes of globalized culture-contact would soon follow.

## SUPPLEMENTARY FIGURES

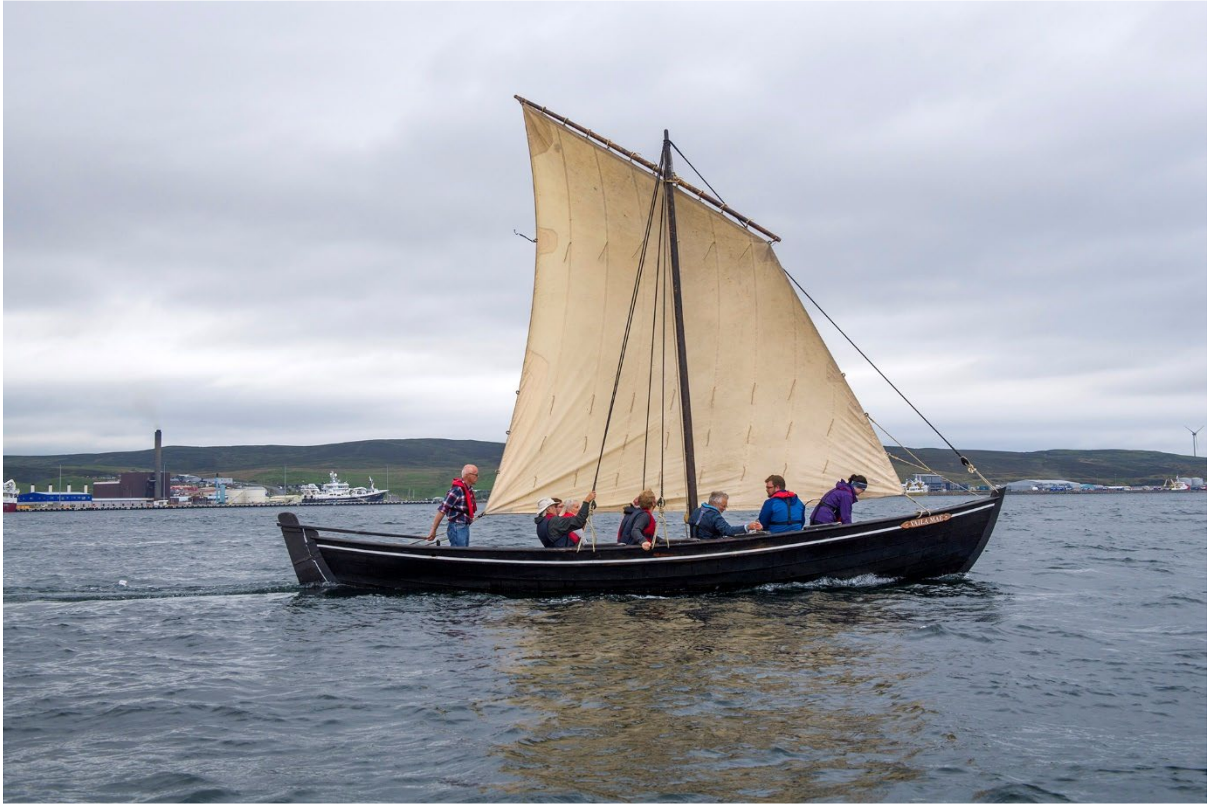

**Supplementary Figure S1. Smaller vessel employed by Greenland Norse.** The Shetland sixareen (pictured) is probably the most similar surviving relative of the smaller six-oared boats used by the Greenland Norse, although such boats would have carried a square rather than a lug sail. Such boats would have been used to harvest local walrus stocks but were not suitable for the High Arctic expeditions to the North Water Polynya. Photo: Dave Donaldson (reproduced with permission).

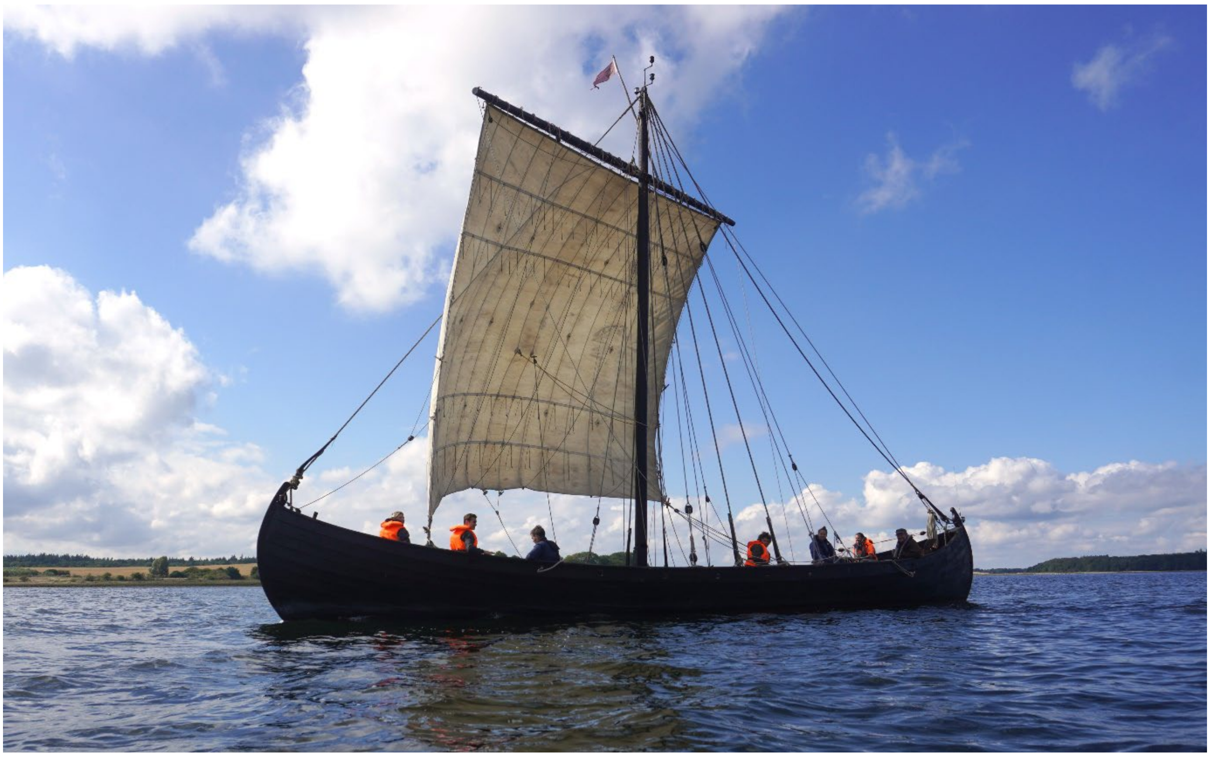

**Supplementary Figure S2. Larger 'expeditionary' vessel employed by Greenland Norse.** This modern example is a compact, clinker-built, square-rigged cargo vessel based on the design of the *Skuldelev 1* wreck, which was built in western Norway c.1030 CE. Norse Greenlanders could only undertake walrus harvesting expeditions to the North Water Polynya in these larger sailing vessels, which were owned by wealthier farmers and elites, adding an important socio-political dimension to Late Period exploitation strategies. Photo: Greer Jarrett.

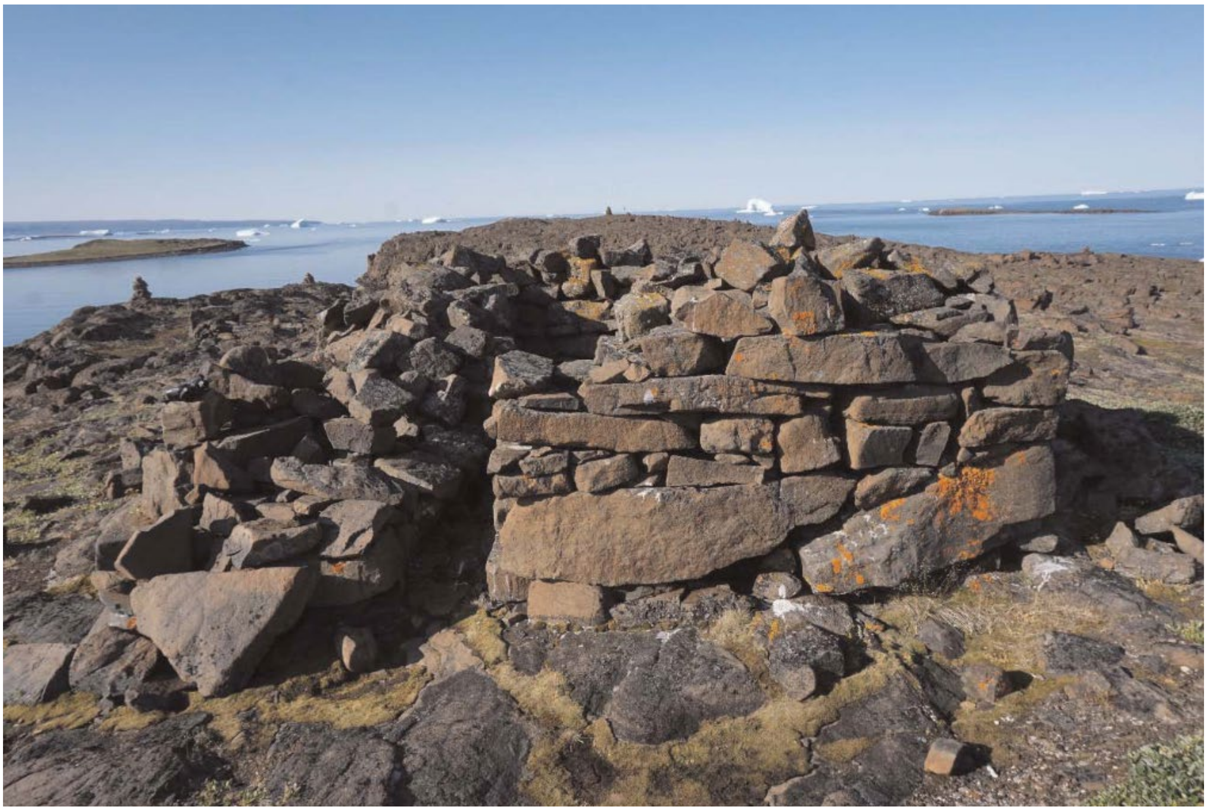

**Supplementary Figure S3**: The Bear Trap (88) and surrounding seascape. This is interpreted as a Greenland Norse storage facility, and is located on the northwestern tip of the Nussuaq Peninsula (Fig. 5). This location offered a sheltered harbour as well as proximity to the outer coast and the open sea, thereby reducing the danger of katabatic winds and becoming trapped by pack ice; the surrounding area also offers ready access to walrus haul-out sites and their shallow feeding grounds. Photo: Matthew Walsh (reproduced with permission).

## SUPPLEMENTARY TABLES

Table S1: Full provenance information: Biological samples.

Table S2: Full provenance information: Cultural samples

Table S3: Estimated rowing distances based on various interpretations of *Grœnlands annál*. For further information, see Supplementary Text.

| | | Distances from the Western Settlement (Nuuk) in days rowing | | | |
|---|---|---|---|---|---|
| Places mentioned in the text (with known locations) | Nautical miles | Jónsson, literal | Jónsson as per Halldórsson (52) | Jónsson as per Nedkvitne (6) | Jónsson as per Ljungqvist (31) |
| Dyrnes (Eastern Settlement) | 327 | ? | 6 | - | 12 |
| Arsuk (Middle Settlement) | 217 | 6 | ? | - | - |
| Lýsufjarðar | ? | 6 | (same as WS) | 6 | - |
| Karlbúða | ? | 12 | 6 | 12 | 12 |
| Bjarneyjar (Godhavn) | 345 | 15 | 9 | 15 | 15 |
| Bjarneyjar (Disko Island) circumference | 215 | 12 | 12 | 12 | 12 |

## REFERENCES AND NOTES

1. T. M. Friesen, C. D. Arnold, The timing of the Thule migration: New dates from the western Canadian Arctic. *Am. Antiq.* **73**, 527–538 (2008).

2. P. Schledermann, A.D. 1000: East Meets West, in *Vikings: The North Atlantic Saga*, W. W. Fitzhugh, E. I. Ward, Eds. (Smithsonian Institution Press, 2000), pp. 189–192.

3. J. Arneborg, The Norse settlements in Greenland, in *The Viking World* (Routledge, 2008), pp. 612–627.

4. T. M. Friesen, S. A. Finkelstein, A. S. Medeiros, Climate variability of the Common Era (AD 1–2000) in the eastern North American Arctic: Impacts on human migrations. *Quat. Int.* **549**, 142–154 (2020).

5. R. McGhee, Contact between native North Americans and the medieval Norse: A review of the evidence. *Am. Antiq.* **49**, 4–26 (1984).

6. A. Nedkvitne, *Norse Greenland: Viking Peasants in the Arctic* (Routledge, 2018).

7. J. Bockstoce, A prehistoric population change in the Bering Strait region. *Polar Record.* **16**, 793–803 (1973).

8. M. S. Murray, Local heroes. The long-term effects of short-term prosperity—An example from the Canadian Arctic. *World Archaeol.* **30**, 466–483 (1999).

9. D. P. Odess, S. Loring, W. W. Fitzhugh, Skraeling: First peoples of Helluland, Markland, and Vinland, in *Vikings: The North Atlantic Saga*, W. Fitzhugh, E. Ward, Eds. (Smithsonian Institution Press, 2000), pp. 193–206.

10. E. J. Ruiz-Puerta, X. Keighley, S. P. A. Desjardins, A. B. Gotfredsen, S. E. Pan, B. Star, S. Boessenkool, J. H. Barrett, M. L. McCarthy, L. W. Andersen, E. W. Born, L. R. Howse, P. Szpak, S. Pálsson, H. J. Malmquist, S. Rufolo, P. D. Jordan, M. T. Olsen, Holocene deglaciation drove rapid genetic diversification of Atlantic walrus. *Proc. Roy. Soc. London, Ser. B, Biol. Sci.* **290**, 1–11 (2023).

11. E. Roesdahl, *Hvalrostand, Elfenben Og Nordboerne i Grønland* (Odense Universitetsforlag, 1995).

12. M. Appelt, E. Damkjar, M. Friesen, Late Dorset, in *The Oxford Handbook of the Prehistoric Arctic*, M. Friesen, O. Mason, Eds. (Oxford University Press, 2016), pp. 783–806.

13. A. B. Gotfredsen, M. Appelt, K. Hastrup, Walrus history around the North Water: Human–animal relations in a long-term perspective. *Ambio* **47**, 193–212 (2018).

14. E. Roesdahl, L'ivoire de morse et les colonies norroises du Groenland. *Proxima Thulé* **3**, 9–48 (1998).

15. E. Roesdahl, Viking art in European churches, in *Viking Trade and Settlement in Continental Western Europe*, I. Skibsted Klæsøe, Ed. (Museum Tusculanum, 2010), pp. 149–164.

16. J. H. Barrett, The exploitation of walrus ivory in medieval Europe, in *The Atlantic Walrus: Multidisciplinary Insights Into Human-Animal Interactions*, X. Keighley, M. T. Olsen, P. Jordan, S. P. Desjardins, Eds. (Elsevier, 2021), pp. 169–196.

17. B. Star, J. H. Barrett, A. T. Gondek, S. Boessenkool, Ancient DNA reveals the chronology of walrus ivory trade from Norse Greenland. *Proc. R. Soc. B* **285**, 1–9 (2018).

18. X. Keighley, S. Pálsson, B. F. Einarsson, A. Petersen, M. Fernández-Coll, P. Jordan, M. T. Olsen, H. J. Malmquist, Disappearance of Icelandic walruses coincided with Norse settlement. *Mol. Biol. Evol.* **36**, 2656–2667 (2019).

19. J. H. Barrett, S. Boessenkool, C. J. Kneale, T. C. O'Connell, B. Star, Ecological globalisation, serial depletion and the medieval trade of walrus rostra. *Quat. Sci. Rev.* **229**, 106122–106115 (2020).

20. K. M. Frei, A. N. Coutu, K. Smiarowski, R. Harrison, C. K. Madsen, J. Arneborg, R. Frei, G. Guðmundsson, S. M. Sindbæk, J. Woollett, S. Hartman, M. Hicks, T. H. McGovern, Was it for walrus? Viking Age settlement and medieval walrus ivory trade in Iceland and Greenland. *World Archaeol.* **47**, 439–466 (2015).

21. C. M. Darwent, G. M. LeMoine, Pre-Inuit walrus use in Arctic Canada and Greenland, c.2500 BCE to 1250 CE, in *The Atlantic Walrus: Multidisciplinary Insights into Human-Animal Interactions*, X. Keighley, M. Tange Olsen, P. D. Jordan, S. P. A. Desjardins, Eds. (Academic Press, 2021), pp. 99–120.

22. S. Desjardins, A. B. Gotfredsen, Subsistence walrus hunting in Inuit Nunangat (Arctic Canada) and Kalaallit Nunaat (Greenland) from the 13th century CE to present, in *The Atlantic Walrus: Multidisciplinary Insights into Human-Animal Interactions*, X. Keighley, M. Tange Olsen, P. D. Jordan, S. P. A. Desjardins, Eds. (Academic Press, 2021), pp. 121–146.

23. A. J. Dugmore, T. H. McGovern, O. Vésteinsson, J. Arneborg, R. Streeter, C. Keller, Cultural adaptation, compounding vulnerabilities and conjunctures in Norse Greenland. *Proc. Natl. Acad. Sci.* **109**, 3658–3663 (2012).

24. P. Schledermann, Ellesmere, in *Vikings: The North Atlantic Saga*, W. W. Fitzhugh, E. I. Ward, Eds. (Smithsonian Institution Press, 2000), pp. 249–256.

25. T. H. McGovern, The Arctic frontier of Norse Greenland, in *Archaeology of Frontiers and Boundaries*, S. W. Green, S. M. Perlman, Eds. (Academic Press, 1985), pp. 275–323.

26. H. C. Gulløv, The nature of contact between native greenlanders and norse. *J. North At.* **1**, 16–24 (2008).

27. K. A. Golding, I. A. Simpson, J. E. Schofield, K. J. Edwards, Norse–Inuit interaction and landscape change in southern Greenland? A geochronological, Pedological, and Palynological investigation. *Geoarchaeology* **26**, 315–345 (2011).

28. J. Arneborg, Contact between Eskimos and Norsemen in Greenland—A review of the evidence, in *Beretning Fra Tolvte Tværfaglige Vikingesymposium*, E. Roesdahl, P. Meulengracht Sørensen, Eds. (Højbjerg, 1993).

29. C. Keller, Furs, fish, and ivory: Medieval Norsemen at the Arctic fringe. *J. North Atl.* **3**, 1–23 (2010).

30. K. A. Seaver, How strange is a stranger? A survey of opportunities for Inuit-European contact in the Davis Strait before 1576, in *Meta Incognita: A Discourse of Discovery. Martin Frobisher's Arctic Expeditions*, T. H. B. Symons, Ed. (Canadian Museum of Civilization, 1999).

31. F. C. Ljungqvist, The significance of remote resource regions for Norse Greenland. *Scripta Islandica* **56**, 13–54 (2005).

32. C. Freitas, K. M. Kovacs, R. A. Ims, M. A. Fedak, C. Lydersen, Deep into the ice: Over-wintering and habitat selection in male Atlantic walruses. *Mar. Ecol. Prog. Ser.* **375**, 247–261 (2009).

33. E. W. Born, L. Ø. Knutsen, Haul-out and diving activity of male Atlantic walruses (*Odobenus rosmarus rosmarus*) in NE Greenland. *J. Zool.* **243**, 381–396 (1997).

34. K. B. Zinglersen, E. Garde, K. Langley, E. R. Mätzler, "Identification of Atlantic Walrus at haul out sites in Greenland using high-resolution satellite images", Technical report (111) Greenland Institute of Natural Resources, Greenland (2020); https://natur.gl/wp-content/uploads/2020/03/GINR_TR_111_RemoteID.pdf.

35. E. Pierce, Walrus hunting and the ivory trade in early Iceland. *Archaeol. Islandica* **7**, 55–63 (2009).

36. B. F. Einarsson, *Landnám Og Landnámsfólk: Saga Af Bæ Og Blóti* (Skrudda, 2015).

37. H. Guðmundsson, *Um haf innan: Vestrænir menn og ízlensk menning á miðöldum* (Háskólaútgáfan, 1997).

38. M. P. Heide-Jørgensen, M.-H. S. Sinding, N. H. Nielsen, A. Rosing-Asvid, R. G. Hansen, Large numbers of marine mammals winter in the North Water polynya. *Polar Biol.* **39**, 1605–1614 (2016).

39. L. Hacquebord, Three centuries of whaling and walrus hunting in Svalbard and its impact on the Arctic ecosystem. *Environ. Hist.* **7**, 169–185 (2001).

40. J. M. Weslawski, L. Hacquebord, Greenland whales and walruses in the Svalbard food web before and after exploitation. *Oceanologia* **1**, 37–56 (2000).

41. B. A. McLeod, T. R. Frasier, Z. Lucas, Assessment of the extirpated Maritimes walrus using morphological and ancient DNA analysis. *PLOS ONE* **9**, 1–14 (2014).

42. G. Eldjárn, J. Godal, *Nordlandsbåten of Åfjordsbåten Bind 1: Båten i Bruk: Segling, Roing, Fisking Og Vedlikehald* (Kjelland, 1988).

43. M. Chivers, M. J. Stratigos, I. Tait, An ethnography of Shetland's oldest boat, the Sixareen Mary LK 981. *Mar.'s Mirror* **105**, 442–460 (2019).

44. J. H. Barrett, N. Khamaiko, G. Ferrari, A. Cuevas, C. Kneale, A. K. Hufthammer, A. H. Pálsdóttir, B. Star, Walruses on the Dnieper: New evidence for the intercontinental trade of Greenlandic ivory in the Middle Ages. *Proc. R. Soc. B* **289**, 1–9 (2022).

45. G. M. LeMoine, C. M. Darwent, The Inglefield Land archaeology project: Introduction and overview. *J. Geogr.* **110**, 279–296 (2010).

46. J. F. Jensen, Greenlandic Dorset, in *Oxford Handbook of the Prehistoric Arctic*, T. M. Friesen, O. K. Mason, Eds. (Oxford University Press, 2016), pp. 737–760.

47. M. H. Smith, K. P. Smith, G. Nilsen, Dorset, Norse, or Thule? Technological transfers, marine mammal contamination, and AMS dating of spun yarn and textiles from the Eastern Canadian Arctic. *J. Archaeol. Sci.* **96**, 162–174 (2018).

48. A. Kuijpers, N. Mikkelsen, S. Ribeiro, M.-S. Seidenkrantz, Impact of medieval fjord hydrography and climate on the western and eastern settlements in Norse Greenland. *J. North Atl.* **601**, 1–13 (2014).

49. L. K. Barlow, J. P. Sadler, A. E. J. Ogilvie, P. C. Buckland, T. Amorosi, J. H. Ingimundarson, P. Skidmore, A. J. Dugmore, T. H. McGovern, Interdisciplinary investigations of the end of the Norse Western Settlement in Greenland. *Holocene* **7**, 489–499 (1997).

50. L. Abrams, Early religious practice in the Greenland Settlement. *J. North Atl.* **201**, 52–65 (2009).

51. K. A. Seaver, Unanswered questions, in *Vikings: The North Atlantic Saga*, W. W. Fitzhugh, E. I. Ward, Eds. (Smithsonian Institution Press, 2000), pp. 268–279.

52..H alldórsson, *Grœnland í miđaldaritum* (Sögufélag, 1978).

53. B. Regal, *The Battle over America's Origin Story: Legends, Amateurs, and Professional Historiographers* (Springer Nature, 2022).

54. M. Kuitems, B. L. Wallace, C. Lindsay, A. Scifo, P. Doeve, K. Jenkins, S. Lindauer, P. Erdil, P. M. Ledger, V. Forbes, C. Vermeeren, R. Friedrich, M. W. Dee, Evidence for European presence in the Americas in ad 1021. *Nature* **601**, 388–391 (2022).

55. A. Þorgilsson, *Íslendingabók : Tilegnet Islands alting 930–1930 af Dansk-Islandsk forbundsfond* (Levin & Munksgaards forlag, 1930).

56. I. Ekrem, L. B. Mortensen, *Historia Norwegie* (Museum Tusculanum Press, 2003).

57. N. Lynnerup, Life and death in Norse Greenland, in *Vikings: The North Atlantic Saga*, W. W. Fitzhugh, Ed. (Smithsonian Institution Press, 2000), pp. 285–294.

58. G. E. Lasher, Y. Axford, Medieval warmth confirmed at the Norse Eastern Settlement in Greenland. *Geology* **47**, 267–270 (2019).

59. J. Arneborg, Greenland and Europe, in *Vikings: The North Atlantic Saga*, W. W. Fitzhugh, E. Ward, Eds. (Smithsonian Institution Press, 2000), pp. 304–318.

60. C. Carøe, S. Gopalakrishnan, L. Vinner, S. S. T. Mak, M. H. S. Sinding, J. A. Samaniego, N. Wales, T. Sicheritz-Pontén, M. T. P. Gilbert, Single-tube library preparation for degraded DNA. *Methods Ecol. Evol.* **9**, 410–419 (2018).

61. U. Arnason, J. A. Adegoke, K. Bodin, E. W. Born, Y. B. Esa, A. Gullberg, M. Nilsson, R. V. Short, X. Xu, A. Janke, Mammalian mitogenomic relationships and the root of the eutherian tree. *Proc. Natl. Acad. Sci. U.S.A.* **99**, 8151–8156 (2002).

62. M. Schubert, L. Ermini, C. D. Sarkissian, H. Jónsson, A. Ginolhac, R. Schaefer, M. D. Martin, R. Fernández, M. Kircher, M. McCue, Characterization of ancient and modern genomes by SNP detection and phylogenomic and metagenomic analysis using PALEOMIX. *Nat. Protoc.* **9**, 1056–1082 (2014).

63. A. Ginolhac, M. Rasmussen, M. T. P. Gilbert, E. Willerslev, L. Orlando, mapDamage: Testing for damage patterns in ancient DNA sequences. *Bioinformatics* **27**, 2153–2155 (2011).

64. M. Schubert, S. Lindgreen, L. Orlando, AdapterRemoval v2: Rapid adapter trimming, identification, and read merging. *BMC. Res. Notes* **9**, 88 (2016).

65. H. Li, B. Handsaker, A. Wysoker, T. Fennell, J. Ruan, N. Homer, G. Marth, G. Abecasis, R. Durbin, 1000 Genome Project Data Processing Subgroup, The sequence alignment/map format and SAMtools. *Bioinformatics* **25**, 2078–2079 (2009).

66. T. S. Korneliussen, A. Albrechtsen, R. Nielsen, ANGSD: Analysis of Next Generation Sequencing Data. *BMC Bioinformatics* **15**, 356 (2014).

67. H. Li, Improving SNP discovery by base alignment quality. *Bioinformatics* **27**, 1157–1158 (2011).

68. L. W. Andersen, E. W. Born, I. Gjertz, Ø. Wiig, L.-E. Holm, C. Bendixen, Population structure and gene flow of the Atlantic walrus (*Odobenus rosmarus rosmarus*) in the eastern Atlantic Arctic based on mitochondrial DNA and microsatellite variation. *Mol. Ecol.* **7**, 1323–1336 (1998).

69. A. J. Drummond, A. Rambaut, BEAST: Bayesian evolutionary analysis by sampling trees. *BMC Evol. Biol.* **7**, 214 (2007).

70. A. Kuijpers, N. Mikkelsen, Geological records of changes in wind regime over south Greenland since the Medieval Warm Period: A tentative reconstruction. *Polar Record.* **45**, 1–8 (2009).

71. W. P. Patterson, K. A. Dietrich, C. Holmden, J. T. Andrews, Two millennia of North Atlantic seasonality and implications for Norse colonies. *Proc. Natl. Acad. Sci.* **107**, 5306–5310 (2010).

72. S. Ribeiro, M. Moros, M. Ellegaard, A. Kuijpers, Climate variability in West Greenland during the past 1500 years: Evidence from a high-resolution marine palynological record from Disko Bay. *Boreas* **41**, 68–83 (2012).

73. B. M. Vinther, P. D. Jones, K. R. Briffa, H. B. Clausen, K. K. Andersen, D. Dahl-Jensen, S. J. Johnsen, Climatic signals in multiple highly resolved stable isotope records from Greenland. *Quat. Sci. Rev.* **29**, 522–538 (2010).

74. *Greenland Pilot: General Information about Greenland* (Danish Geodata Agency, 2020).

75. *Greenland Pilot: Sailing Directions for West Greenland* (Danish Geodata Agency, 2018).

76. A. Mønsted, M. Appelt, A. B. Gotfredsen, C. Houmard, A. Zazzo, S. Cersoy, O. Tombret, B. Grønnow, An early Inuit workshop at a *Qassi*, a men's house, Nuulliit, Northwest Greenland. *Arctic Anthro.* **59**, 3–38 (2023).

77. A. Roussell Sandnes and the neighbouring farms, in *Meddelelser Om Grønland*, C. A. Reitzels, Ed. (C. A. Reitzels forlag, 1936).

78. I. A. Morrison, "Aspects of Viking small craft in the light of Shetland practice" in *Scandinavian Shetland: An Ongoing Tradition*?, J. R. Baldwin, Ed. (Scottish society for northern studies, Edinburgh, 1978), pp. 57–75.

79. A. E. Christensen, The "Big Ship" of Bryggen in Bergen: What can it tell us? *Deutsches Schiffahrtsarchiv* **25**, 87–95 (2002).

80. P. Nørlund, M. Stenberger, *Brattahlid: Researches into Norse Culture in Greenland* (Reitzel, 1934).

81. C. Madsen, *Pastoral Settlement, Farming, and Hierarchy in Norse Vatnahverfi, South Greenland*, (Université de Copenhague, 2014).

82. O. Færøyvik, *Inshore Craft of Norway* (Conway Maritime Press, 1979).

83. W. H. Carter, *A Viking Voyage: In Which an Unlikely Crew Attempts an Epic Journey to the New World* (Ballantine Books, 2001).

84. R. Morcken, *Veien Mot Nord: Vikingetidens Distansetabell Langs Den Norske Kyst Fra Svenskegrensen Til Hvitehavet* (Bergens sjøfartsmuseum, 1978.

85. J. Smiley, R. Kellogg, *The Sagas of the Icelanders: A Selection* (Penguin, 2001).

86. M. Vinner, A viking-ship off cape farewell 1984, in *Shipshape. Essays for Ole Crumlin-Pedersen on the Occasion of His 60th Anniversary February 24th 1995* (Vikingeskibshallen i Roskilde, 1995), pp. 289–304.

87. B. L. Wallace, An archaeologist's Interpretation of the Vinland Sagas, in *Vikings: The North Atlantic Saga*, W. W. Fitzhugh, E. I. Ward, Eds. (Smithsonian Institution Press, 2000), pp. 228–231.

88. M. J. Walsh, D. F. Carlson, P. Tejsner, S. Thomsen, The bear trap: Reinvestigation of a unique stone structure on the northwest tip of the Nuussuaq Peninsula Greenland. *Arctic Anthro.* **58**, 200–217 (2023).

89. B. Grønnow, M. Appelt, A. B. Gotfredsen, M. Myrup, *Arkæologiske og arkæo-zoologiske undersøgelser på bopladserne Nuulliit og Uummannaq (Avanersuaq) – en del af NOW Projektet, 2015* (National Museum of Denmark, 2016).

90. J. McDermott, *Martin Frobisher: Elizabethan Privateer* (Yale University Press, 2001).

91. P. D. Sutherland, The question of contact between Dorset Palaeo-Eskimos and early Europeans in the Eastern Arctic, in *The Northern World, AD 900–1400*, H. D. G. Maschner, O. K. Mason, R. McGhee, Eds. (University of Utah Press, 2009), pp. 270–299.

92. P. D. Sutherland, P. H. Thompson, P. A. Hunt, Evidence of early metalworking in Arctic Canada. *Geoarchaeology* **30**, 74–78 (2015).

93. H. C. Gulløv, *From Middle Ages to Colonial Times: Archaeological and Ethnohistorical Studies of the Thule Culture in South West Greenland 1300–1800 AD* (Commission for Scientific Research in Greenland, 1997).

94. D. Sabo, G. Sabo, A possible Thule carving of a viking from Baffin Island, N.W.T. *Can. J. Archaeol.* **2**, 33–42 (1978).

95. P. Schledermann, Notes on Norse finds from the east coast of Ellesmere Island, N.W.T. *Arctic* **33**, 454–463 (1980).
